# Role of ALDH1A1 and HTRA2 expression in CCL2/CCR2-mediated breast cancer cell growth and invasion

**DOI:** 10.1242/bio.040873

**Published:** 2019-06-17

**Authors:** Qingting Hu, Megan Myers, Wei Fang, Min Yao, Gage Brummer, Justin Hawj, Curtis Smart, Cory Berkland, Nikki Cheng

**Affiliations:** 1 Department of Pathology and Laboratory Medicine, University of Kansas Medical Center, Kansas City, KS 66160, USA; 2 Department of Pharmaceutical Chemistry, University of Kansas, Lawrence, KS 66047, USA; 3 Department of Cancer Biology, University of Kansas Medical Center, Kansas City, KS 66160, USA

**Keywords:** CCL2, CCR2, Breast cancer, 3D culture, Cell invasion, ALDH1A1, HTRA2

## Abstract

Chemokines mediate immune cell trafficking during tissue development, wound healing and infection. The chemokine CCL2 is best known to regulate macrophage recruitment during wound healing, infection and inflammatory diseases. While the importance of CCL2/CCR2 signaling in macrophages during cancer progression is well documented, we recently showed that CCL2-mediated breast cancer progression depends on CCR2 expression in carcinoma cells. Using 3D Matrigel: Collagen cultures of SUM225 and DCIS.com breast cancer cells, this study characterized the mechanisms of CCL2/CCR2 signaling in cell growth and invasion. SUM225 cells, which expressed lower levels of CCR2 than DCIS.com cells, formed symmetrical spheroids in Matrigel: Collagen, and were not responsive to CCL2 treatment. DCIS.com cells formed asymmetric cell clusters in Matrigel: Collagen. CCL2 treatment increased growth, decreased expression of E-cadherin and increased TWIST1 expression. CCR2 overexpression in SUM225 cells increased responsiveness to CCL2 treatment, enhancing growth and invasion. These phenotypes corresponded to increased expression of Aldehyde Dehydrogenase 1A1 (ALDH1A1) and decreased expression of the mitochondrial serine protease HTRA2. CCR2 deficiency in DCIS.com cells inhibited CCL2-mediated growth and invasion, corresponding to decreased ALDH1A1 expression and increased HTRA2 expression. ALDH1A1 and HTRA2 expression were modulated in CCR2-deficient and CCR2-overexpressing cell lines. We found that ALDH1A1 and HTRA2 regulates CCR2-mediated breast cancer cell growth and cellular invasion in a CCL2/CCR2 context-dependent manner. These data provide novel insight on the mechanisms of chemokine signaling in breast cancer cell growth and invasion, with important implications on targeted therapeutics for anti-cancer treatment.

This article has an associated First Person interview with the first author of the paper.

## Abstract (precis)

**Summary:** Chemokines are known to regulate immune cell recruitment during inflammation. This report characterizes novel molecular mechanisms through which CCL2/CCR2 chemokine signaling in breast cancer cells regulates growth and invasion.

## INTRODUCTION

Chemokines are small soluble proteins (8 kda) that regulate cellular homing and recruitment to tissues through formation of concentration gradients. They are highly conserved among mammals, and mediate immune cell trafficking and angiogenesis during tissue development, wound healing and infection (Proost et al., 2017; Rees et al., 2015; Ridiandries et al., 2016). More than 50 chemokine ligands and 25 chemokine receptors have been identified, and are categorized into several classes depending on the composition of a conserved cysteine motif at the N terminus: C-C, C-X-C and CX3C, in which the X is a non-cysteine amino acid residue (Borroni et al., 2018; Lacalle et al., 2017; Yao et al., 2016a). CCL2 (MCP-1) belongs to the C-C class of chemokines, and is a critical regulator of macrophage recruitment during wound healing, infection and chronic inflammatory diseases such as rheumatoid arthritis (De Paepe et al., 2008; Koelink et al., 2009). While CCL2 is capable of binding multiple receptors, it binds with highest affinity to CCR2 (Bonini and Steiner, 1997; Monteclaro and Charo, 1996). CCL2/CCR2 signaling in macrophages leads to increased chemotaxis and cellular adhesion through activation of G proteins and signaling through p42/44MAPK, Phospho-Lipase C gamma and Protein Kinase C pathways (Ashida et al., 2001). Mice exhibiting knockout of CCL2 or CCR2 show defects in macrophage recruitment during bacterial infection, macular degeneration or autoimmune encephalitis (Boring et al., 1997; Huang et al., 2001; Kurihara et al., 1997). The lack of compensatory upregulation of chemokine ligands or receptors indicates unique biological functions for CCL2/CCR2 signaling during inflammation.

CCL2 and CCR2 expression are chronically overexpressed in multiple cancer types including: glioblastoma, prostate, colon and breast cancer (Baier et al., 2005; Chavey et al., 2007; Leung et al., 1997; Tsaur et al., 2015). In breast cancer patients, elevated levels of CCL2 have been detected in blood serum (Lebrecht et al., 2004). Furthermore, increased CCL2 protein expression in breast tumor tissues are associated with macrophage levels, and correlate with tumor grade and poor patient prognosis (Fujimoto et al., 2009; Saji et al., 2001; Ueno et al., 2000; Yao et al., 2016b). In animal models of breast cancer, stable expression of CCL2 shRNAs in breast tumor xenografts or treatment of primary tumors with CCL2 neutralizing antibodies leads to decreased primary tumor growth and systemic metastasis, correlating with decreased recruitment of M2 polarized macrophages to tissues (Fujimoto et al., 2009; Hembruff et al., 2010; Qian et al., 2011). These studies demonstrate that CCL2 promotes breast cancer progression in part through recruitment of macrophages to the primary tumor.

While the importance of CCL2/CCR2 signaling in macrophages during cancer progression is well documented, we recently showed that CCL2-mediated breast cancer progression depends on CCR2 expression in carcinoma cells. By immunostaining, CCR2 protein was found to be overexpressed in breast carcinoma tissues, and datamining analysis revealed that RNA levels correlated with decreased distant metastasis free survival (Brummer et al., 2018; Fang et al., 2012). Flow cytometry analysis of breast cancer cell lines demonstrated that CCR2 protein expression correlated with invasive potential (Fang et al., 2012). In particular, highly invasive DCIS.com breast cancer cells expressed higher levels of CCR2 than lowly invasive SUM225 cells. shRNA knockdown of CCR2 in DCIS.com breast cancer cells inhibited formation of invasive breast carcinomas in animal models. CCR2 overexpression in SUM225 breast cancer cells enhanced formation of breast carcinomas (Brummer et al., 2018). These studies have demonstrated a unique role for CCR2 expression in epithelial cells in regulating invasive progression of breast carcinomas. Yet, the mechanisms through CCL2/CCR2 signaling in epithelial cells facilitate these disease processes remain poorly understood.

Using 3D Matrigel: Collagen cultures of SUM225 and DCIS.com breast cancer cells, this study characterized the mechanisms through which CCL2/CCR2 signaling regulated cell growth and invasion. SUM225 cells formed symmetrical spheroids in Matrigel: Collagen, were lowly invasive, and were not responsive to CCL2 recombinant protein. DCIS.com cells formed asymmetric cell clusters in Matrigel: Collagen. CCL2 treatment increased growth and increased expression of TWIST, a mesenchymal marker and decreased expression of E-cadherin, an epithelial marker. CCR2 overexpression in SUM225 cells increased responsiveness to CCL2 treatment, resulting in increased growth, cellular invasion and increased expression of TWIST and decreased expression of E-cadherin. These pro-tumorigenic phenotypes corresponded to increased expression of Aldehyde Dehydrogenase 1A1 (ALDH1A1), and decreased expression of the mitochondrial serine protease HTRA2. CRISPR knockout or shRNA knockdown of CCR2 in DCIS.com cells inhibited CCL2-mediated growth, EMT and invasion, corresponding to decreased ALDHA1 expression and increased HTRA2 expression. We modulated ALDH1A1 and HTRA2 expression in CCR2 deficient and breast cancer cell lines with induced or endogenous CCR2 overexpression. We found that ALDH1A1 and HTRA2 regulates CCR2-mediated breast cancer cell growth and cellular invasion in a CCL2/CCR2 context-dependent manner. In summary, CCL2/CCR2 chemokine signaling regulates breast cancer cell growth and invasion through increased ALDH1A1 expression and suppression of HTRA2. These data provide novel insight in the mechanisms of chemokine signaling in breast cancer cell growth and invasion, with important implications on development of targeted therapeutics for anti-cancer treatment.

## RESULTS

### CCL2 enhances growth and invasion of DCIS.com but not SUM225 cells

**Fig. 1.**
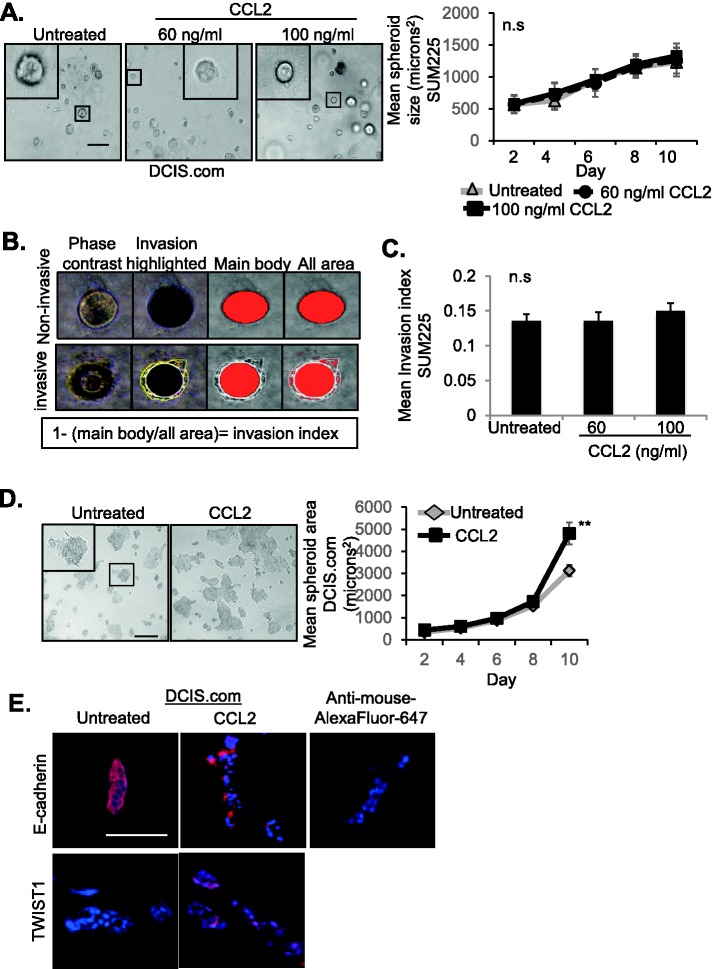
**Responsiveness of SUM225 and DCIS.com breast** **cancer cells** **to CCL2 treatment in 3D cultures.** (A–C) SUM225 breast cancer cells were cultured in Matrigel: Collagen, in the presence or absence of recombinant CCL2 (60 or 100 ng/ml), and analyzed for changes in spheroid size over time for up to 10 days. Representative images of spheroids at day 10 are shown with magnified inset. Spheroid size was measured using ImageJ (A). Invasion index was calculated by dividing total area by main body area of each spheroid and subtracting from 1 (B). Invasion index was determined for SUM225 3D cultures in the presence or absence of CCL2 treatment (C). Spheroid size and invasion index were normalized to spheroid number. Mean number of spheroids analyzed per group±s.e.m: untreated, 170±58; 60 ng/ml CCL2, 159±35; 100 ng/ml CCL2, 183±32. (D,E) DCIS.com cells were embedded in Matrigel: Collagen, treated with 60 ng/ml CCL2, and analyzed for growth over time for up to 10 days (D). Representative images of asymmetric cell clusters are shown with magnified inset. 3D cell culture sections were immunostained for expression of E-Cadherin or TWIST1, with DAPI counterstain at day 10 (E). Experimental groups were plated in triplicate and experiments were performed three times. Two slides containing three sections each were subject to immunostaining. Mean number of spheroids analyzed per group±s.e.m.: untreated, 267±34; CCL2, 239±54. Statistical analysis was performed using two-tailed *t*-test (D) or one-way ANOVA with Bonferroni post-hoc comparison (A,C). **P<0.01; n.s, not significant. Scale bars: 100 µm. Mean±s.e.m. is shown.

Tumor malignancy is characterized by loss of tissue-defining structures, and can be modeled by 3D cultures (Debnath et al., 2002; Herrmann et al., 2014; Krause et al., 2008; Li and Lu, 2011). Normal or lowly invasive cells form symmetrical hollow spheroids, while highly invasive cancer cells form asymmetrical cell clusters with no discernable shape. To elucidate the mechanisms through which CCL2/CCR2 signaling regulated breast cancer cell growth and invasion, we first analyzed the effects of CCL2 treatment on DCIS.com and SUM225 breast cancer cell growth and invasion in 3D Matrigel: Collagen cultures. SUM225 cells, which expressed lower levels of CCR2 than DCIS.com cells (Brummer et al., 2018), formed symmetrical spheroids in Matrigel: Collagen. Spheroid size was not affected by CCL2 treatment at increasing concentrations (Fig. 1A). As SUM225 cell clusters formed visible invasive protrusions, the effects of CCL2 treatment on cellular invasion in 3D cultures were quantified. The invasion index (Cisneros Castillo et al., 2016) was calculated by dividing total area by main body area of each spheroid and subtracting from 1 (Fig. 1B). CCL2 treatment did not affect invasiveness of SUM225 cells in 3D cultures (Fig. 1C). In contrast, DCIS.com cells formed asymmetrical cell clusters, which spread out in Matrigel: Collagen. These cell clusters significantly increased in size over time in response to CCL2 treatment, correlating with decreased E-cadherin expression, an epithelial marker and increased TWIST1 expression, a mesenchymal marker (Fig. 1D–E) (Soini et al., 2011; Zheng and Kang, 2014).

### CCR2 expression is required for CCL2-mediated breast cancer cell growth and invasion

**Fig. 2.**
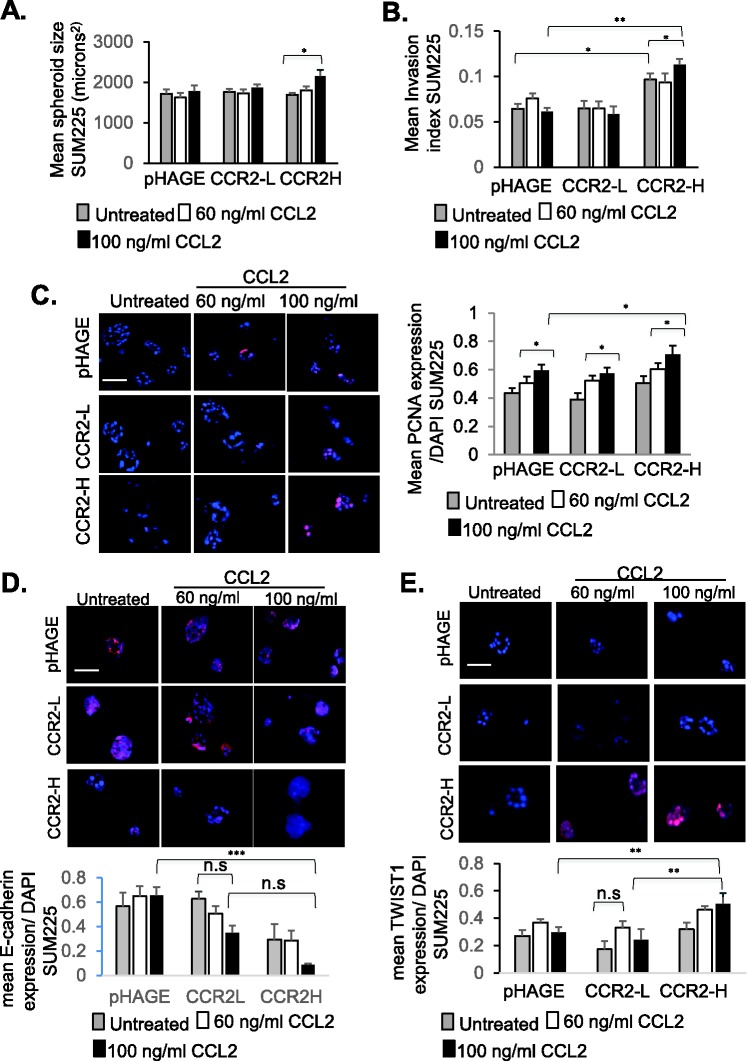
**CCR2 overexpression in** **SUM225 breast cancer** **cells enhances growth and invasion.** (A,B) pHAGE control or CCR2-overexpressing SUM225 cells (CCR2-L, CCR2-H) were cultured in 3D Matrigel: Collagen for up to 10 days, and measured for (A) spheroid size and (B) invasion index. Spheroid size and invasion index were normalized to spheroid number. (C–E) SUM225 cultures were immunofluorescence stained for (C) PCNA, (D) E-Cadherin or (E) TWIST1 expression. Expression was quantified by ImageJ, and normalized to DAPI. Experimental groups were plated in triplicate and experiments were performed three times. Two slides containing three sections each were subject to immunostaining. Mean number of spheroids analyzed per group±s.e.m.: pHAGE untreated, 143±12; pHAGE+60 ng/ml CCL2, 196±23; pHAGE+100 ng/ml CCL2, 172±12; CCR2-L untreated, 161±27; CCR2-L+60 ng/ml CCL2, 165±23; CCR2-L+100 ng/ml CCL2, 220±16; CCR2-H untreated, 138±29; CCR2-H+60 ng/ml CCL2, 132±5; CCR2-H+100 ng/ml CCL2, 158±21. Statistical analysis was performed using one-way ANOVA with Bonferroni post-hoc comparison. *P<0.05, **P<0.01, ***P<0.001; n.s, not significant. Scale bars: 100 µm. Mean±s.e.m. is shown.

We next determined whether increased CCR2 expression in SUM225 cells would enhance responsiveness to CCL2 treatment. Two cell lines were generated to overexpress CCR2, one showing 22.1% positive expressing cells (CCR2-L) and the second one showing 41.5% positive expressing cells (CCR2-H), compared to 8.5% CCR2 positive in pHAGE vehicle control cells (Brummer et al., 2018). Compared to pHAGE control cells, CCR2-L and CCR2-H cultured alone did not show changes in spheroid size in Matrigel: Collagen (Fig. 2A). However, CCR2-H cells cultured alone showed a higher invasion index, compared to pHAGE and CCR2-L cells (Fig. 2B), and was associated with higher CCL2 levels compared to pHAGE control (Fig. S1). CCL2 treatment at 100 ng/ml, but not 60 ng/ml, further enhanced spheroid size and increased the invasion index of CCR-H cells. As a complementary approach, cellular invasion was quantified by Matrigel transwell invasion assay. Similarly to 3D cultures, 100 ng/ml CCL2 enhanced transwell invasion of CCR2-H cells (Fig. S2). In contrast to 3D cultures, we noted that untreated CCR2-H cells did not show changes in transwell invasion, possibly reflecting differences in matrix interactions in a 3D environment versus a monolayer with SUM225 cells. Overall, these data indicate that CCL2 treatment of CCR2-H cells increased spheroid size and invasiveness in a dose-dependent manner.

To determine whether CCL2/CCR2-mediated changes in spheroid size and invasion were related to cell proliferation, survival and EMT, immunofluorescence staining was performed on 3D cultures. CCR2 overexpression and CCL2 treatment of SUM225 cells did not affect cleaved caspase-3 expression (Fig. S3), indicating no changes in apoptosis (Parton et al., 2002). 100 ng/ml CCL2 treatment of CCR2-H cells increased expression of PCNA (Fig. 2C), a cell proliferation marker (van Dierendonck et al., 1991). CCR2-H cells showed a partial decrease in E-cadherin expression, which was further decreased with 100 ng/ml CCL2 treatment (Fig. 2D). CCR2-H cells showed increased TWIST1 expression when treated with 60 ng/ml or 100 ng/ml CCL2 (Fig. 2E). Interestingly, 100 ng/ml CCL2 treatment of pHAGE and CCR2-L cells increased PCNA expression, although increased PCNA levels were not sufficient to significantly affect overall spheroid size in these groups. pHAGE and CCR2-L still expressed CCR2 but in a smaller percentage of cells than CCR2-H cells (Brummer et al., 2018). It is possible that CCR2 expression in pHAGE and CCR2-L cells enabled responsiveness to CCL2 and increased PCNA staining (Fig. 2C), but was not sufficient enough to contribute to spheroid growth over time. Alternatively, the cell cycle of SUM225 cells could have been so slow such that the increase in PCNA expression would not be reflected in an immediate increase in spheroid size. In summary, these data indicate that increased CCL2/CCR2 signaling in SUM225 breast cancer cells promotes cell proliferation, and invasion associated with increased TWIST1 and decreased E-cadherin. Given that CCR2-H but not CCR2-L cells showed changes in cellular phenotype, these data indicate that levels of CCR2 expression in SUM225 cells determine CCL2 responsiveness.

To further the significance of CCR2 expression to breast cancer cell growth and invasion, we induced CCR2 expression in established 3D cultures. TAT cell penetrating peptides form non-covalent crosslinks to plasmid DNA in the presence of CaCl_2_. These complexes form nanoparticles that efficiently penetrate cells and tissues to modulate gene expression (Baoum et al., 2012, 2009). SUM225 cells were first cultured in Matrigel: Collagen for 2 days, and then transfected with TAT cell penetrating peptides complexed to a vehicle control plasmid (Ca-TAT/pHAGE) or CCR2 overexpression plasmid (Ca-TAT/CCR2). Delivery of Ca-TAT/CCR2 complexes, but not Ca-TAT/pHAGE controls, visibly increased CCR2 expression in SUM225 cells (Fig. S4A). Induction of CCR2 expression increased spheroid size over time (Fig. S4B), which was associated with increased cellular invasion, not cell proliferation (Fig. S4C,D). These data are consistent with the phenotypes observed with SUM225 CCR2-H cells. In summary, these data indicate that CCR2 overexpression in established SUM225 spheroid cultures enhances breast cancer cell invasion.

**Fig. 3.**
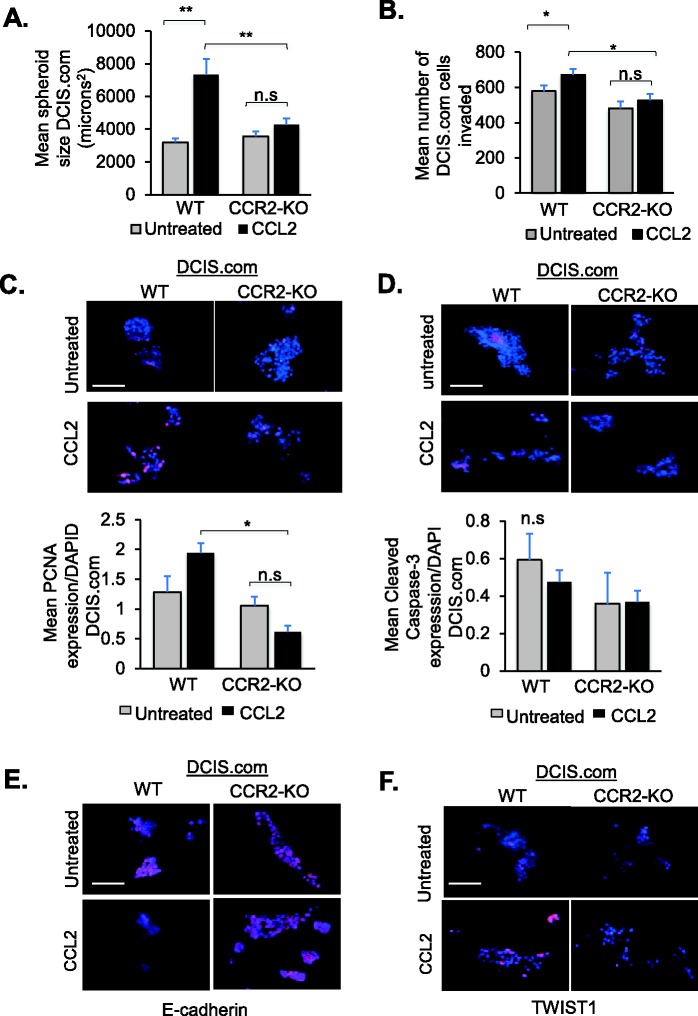
**CRISPR gene ablation of CCR2** **in DCIS.com cells inhibits growth and invasion.** (A) DCIS.com cells expressing wild-type CCR2 (WT) or CRISPR knocked out for CCR2 (CCR2-KO) were embedded in Matrigel: Collagen for up to 10 days and analyzed for changes in spheroid size. (B) DCIS.com cells were treated with 60 ng/ml CCL2 and invasion was assessed by Matrigel transwell assay for 24 h. (C–F) DCIS.com 3D cultures were analyzed for expression of PCNA (C), Cleaved Caspase-3 (D), E-cadherin (E) or TWIST1 (F) with DAPI counterstain. Spheroid size was normalized to spheroid number. Expression was quantified by ImageJ, and normalized to DAPI. Experimental groups were plated in triplicate. Experiments were performed three times. Two slides containing three sections each were subject to immunostaining. Mean number of spheroids analyzed per group±s.e.m.: WT untreated, 194±12; WT±CCL2, 188±19; CCR2-KO untreated, 195±67; CCR2-KO±CCL2, 268±21. Data are representative of four experiments. Statistical analysis was performed using one-way ANOVA with Bonferroni post-hoc comparison. *P<0.05, **P<0.01; n.s, not significant. Scale bars: 100 µm. Mean+s.e.m. is shown.

As a complementary approach, we examined the effects of CCR2 deficiency on DCIS.com growth and invasion. In previous studies, we had ablated CCR2 gene expression in DCIS.com cells by CRISPR (CCR2-KO), which inhibited formation of invasive ductal carcinomas (Brummer et al., 2018). In wild-type (WT) DCIS.com cells, 35.1% of cells were positive for CCR2 expression. 12.3% of cells in CCR2-KO cells were CCR2 positive (Brummer et al., 2018). In 3D cultures, CCR2 knockout alone did not affect the size of DCIS.com cell clusters but inhibited CCL2 induction of cell growth (Fig. 3A). As invasion index was difficult to assess with the asymmetric cell cluster formation of DCIS.com cells in 3D cultures; invasion was quantified by transwell assay. CCR2 knockout alone did not significantly affect transwell invasion, but inhibited CCL2-mediated invasion (Fig. 3B). Biomarker staining analysis indicated that CCR2-KO inhibited CCL2 induction of PCNA expression in WT DCIS.com cells (Fig. 3C). Cleaved caspase-3 expression was not affected by CCR2-KO or CCL2 treatment (Fig. 3D). These data indicate that CCL2 enhances DCIS.com cell proliferation but not apoptosis in a CCR2-dependent manner. Analysis of EMT markers indicated that CCL2 inhibited E-cadherin expression in WT cells. CCR2-KO prevented CCL2 suppression of E-cadherin expression (Fig. 3E). CCL2 increased TWIST1 expression in WT cells, which was inhibited with CCR2-KO (Fig. 3F). In summary, these data indicate that CCL2/CCR2 signaling enhances DCIS.com cell proliferation and invasion associated with increased TWIST1 and decreased E-cadherin.

To validate the effects of CCR2-KO, we examined DCIS.com cells that stably expressed CCR2 shRNAs (CCR2-KD). Compared to cells expressing control shRNAs, CCR2-KD cells showed a 50% decrease in CCR2 expression, compared to cells expressing control shRNAs, corresponding to decreased formation of invasive ductal carcinomas in mice (Brummer et al., 2018). CCR2-KD inhibited CCL2-mediated cell growth and invasion (Fig. S5A,B), associated with decreased PCNA expression, but there were no changes in Cleaved Caspase-3 expression (Fig. S5C,D). CCL2 inhibited E-cadherin expression in control cells. CCR2-KD prevented CCL2 suppression of E-Cadherin expression (Fig. S5E). CCR2-KD suppressed CCL2-mediated TWIST1 expression in DCIS.com cells (Fig. S5F). These phenotypes are similar to CRISPR gene ablation of CCR2.

### CCL2/CCR2-mediated breast cancer cell growth and invasion are associated with increased ALDH1A1 and decreased HTRA2 expression

**Fig. 4.**
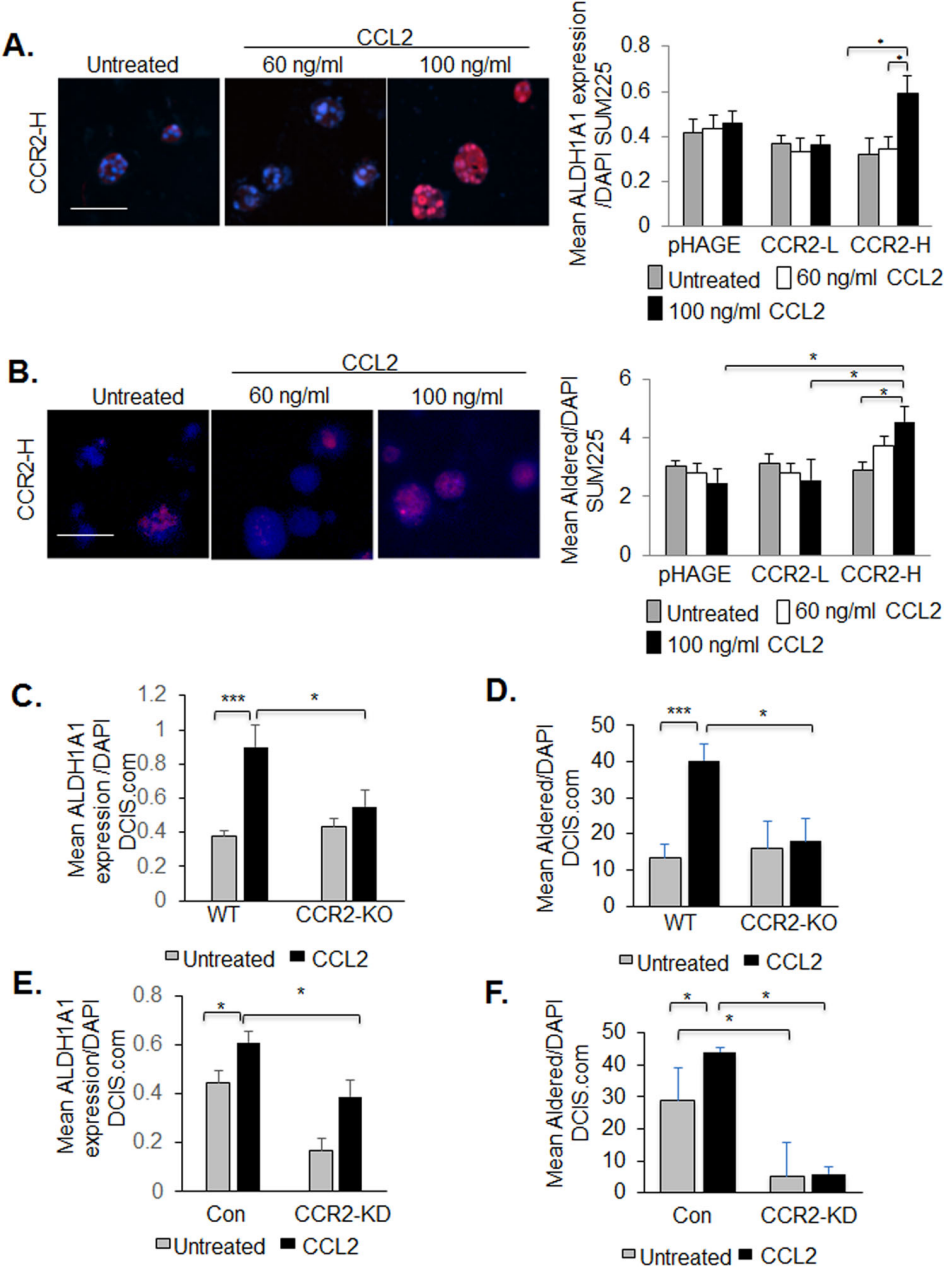
**CCR2 positively regulates ALDH1A1 expression in SUM225 and DCIS.com breast cancer cells**. (A,B) 3D cultures of pHAGE, CCR2-L or CCR2-H SUM225 cells were treated with or without 60 or 100 ng/ml CCL2 and analyzed for ALDH1A1 expression by immunofluorescence staining (A) or activity by Aldered assay (B). (C,D) 3D cultures of WT or CCR2-KO DCIS.com breast cancer cells were treated with or without 60 ng/ml CCL2 and analyzed for ALDH1A1 expression by immunofluorescence staining (C) or activity by Aldered assay (D). (E,F) 3D cultures of control (Con) or CCR2-KD DCIS.com cells were treated with or without 60 ng/ml CCL2 and analyzed for ALDH1A1 expression by immunofluorescence staining (E) or activity by Aldered assay (F). Expression was quantified by ImageJ, and normalized to DAPI. Experimental groups were plated in triplicate and repeated three times. Two slides containing three sections each were subject to immunostaining. Statistical analysis was performed using one-way ANOVA with Bonferroni post-hoc comparison. *P<0.05, ***P<0.001. Scale bars: 100 µm. Mean+s.e.m. is shown.

To determine the molecular mechanisms through which CCL2/CCR2 signaling regulates breast cancer cell growth and invasion, we examined for expression of downstream factors. ALDH1A1 is a metabolic enzyme that functions as a pro-invasive factor and stem cell marker (Ginestier et al., 2007; Sapudom et al., 2015), and positively corresponded to CCR2 expression in breast carcinomas (Brummer et al., 2018). In 3D cultures, CCR2 overexpression alone in SUM225 cells (CCR2-L and CCR2-H) did not affect ALDH1A1 expression or activity compared to pHAGE control cells. CCL2 treatment of CCR2-H cells enhanced ALDH1A1 expression and activity, compared to pHAGE or CCR2-L cells treated with CCL2 (Fig. 4A,B). In DCIS.com WT cells, CCL2 increased ALDH1A1 expression and activity, which was inhibited with CCR2-KO (Fig. 4C,D). Similarly, CCL2 increased ALDH1A1 expression and activity in DCIS.com expressing control shRNA cells which were significantly decreased with CCR2-KD (Fig. 4E,F). These data indicate that CCL2/CCR2 signaling positively regulates ALDH1A1 expression and activity in breast cancer cells.

**Fig. 5.**
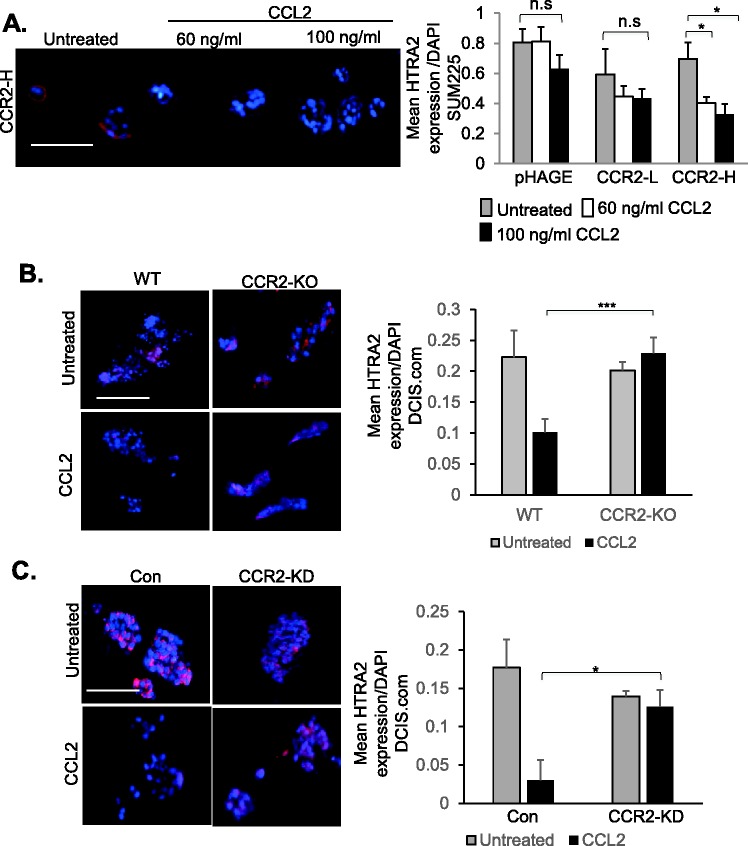
**CCR2 negatively regulates HTRA2 expression in SUM225 and DCIS.com breast cancer cells.** (A) 3D cultures of pHAGE, CCR2-L or CCR2-H SUM225 cells were treated with or without 60 or 100 ng/ml CCL2 and analyzed for HTRA2 expression by immunofluorescence staining. (B,C) WT or CCR2-KO DCIS.com cells (B), Con shRNA or CCR2-KD DCIS.com cells (C) were cultured in 3D matrices in the presence of absence of 100 ng/ml CCL2 and analyzed for HTRA2 expression by immunofluorescence staining. Expression was quantified by ImageJ and normalized to DAPI. Experimental groups were plated in triplicate and experiments were performed three times. Two slides containing three sections each were subject to immunostaining. Statistical analysis was performed using one-way ANOVA with Bonferroni post-hoc comparison. *P<0.05, ***P<0.001; n.s, not significant. Scale bars: 100 µm. Mean+s.e.m. is shown.

Previous studies showed that HTRA2, a pro-apoptotic mitochondrial serine protease (Xu et al., 2012) inversely corresponded to CCR2 expression in breast cancer (Brummer et al., 2018). In SUM225 cells, CCR2 overexpression alone (CCR2-L, CCR2-H) did not significantly affect HTRA2 expression (Fig. 5A). CCL2 treatment at 60 ng/ml and 100 ng/ml significantly decreased HTRA2 expression in CCR2-H, but not CCLR2-L or pHAGE control cells. In DCIS.com cells, CCL2 treatment decreased HTRA2 expression in WT and control shRNA expressing cells. CCR2-KO or CCR2-KD prevented CCL2 suppression of HTRA2 expression (Fig. 5B,C). These data indicate that CCL2/CCR2 signaling negatively regulates HTRA2 expression in breast cancer cells.

### ALDH1A1 positively regulates CCR2-mediated breast cancer cell growth and invasion in a CCL2/CCR2 context-dependent manner

**Fig. 6.**
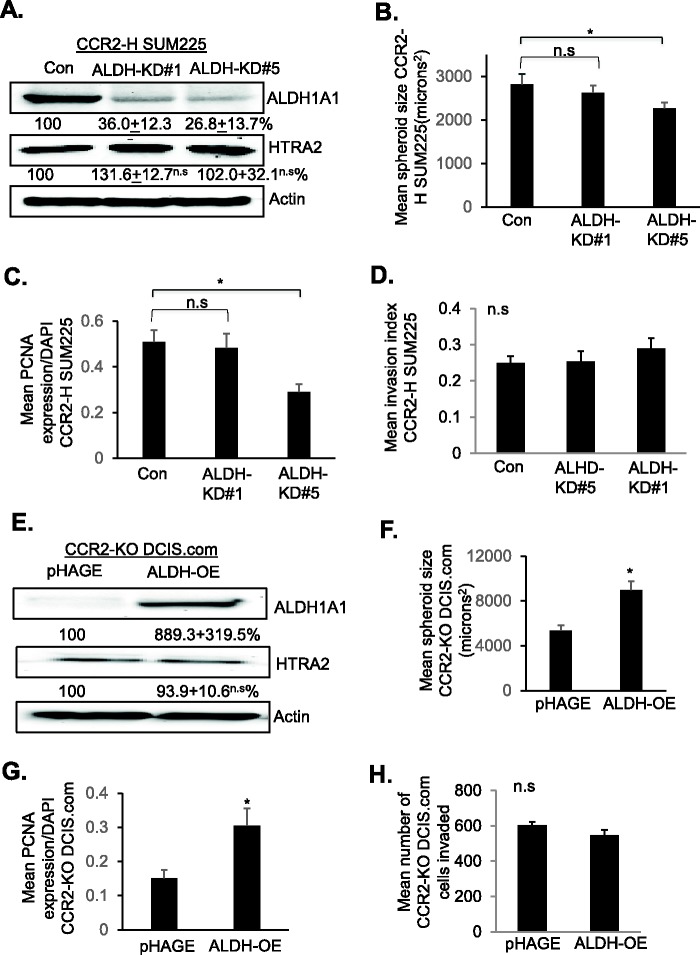
**ALDH1A1 overexpression or** **knockdown affects CCR2-H SUM225 and CCR2-KO DCIS.com breast cancer cell growth but not invasion.** (A–D) CCR2-H SUM225 cells expressing Con shRNAs or shRNAs to ALDH1A1 (ALDH-KD#1, ALDH-KD#5) were analyzed for ALDH1A1 and HTRA2 expression by immunoblot (A), spheroid size in 3D cultures (B), PCNA expression (C) or invasion index (D). Mean number of spheroids analyzed per group±s.e.m.: Con, 197±18; ALDH-KD#1, 190±10; ALDH-KD#5, 196±87. (E–H) CCR2-KO DCIS.com cells expressing vehicle control (pHAGE) or ALDH1A1 (ALDH-OE) were analyzed for: expression of ALDH1A1 and HTRA2 by immunoblot (E), spheroid size in 3D cultures (F), PCNA expression (G) or transwell invasion (H). Protein levels in immunoblots were measured by densitometry. Expression levels were normalized to control group. Representative blots of three experiments are shown. Spheroid size and invasion index were normalized to spheroid number. Experimental groups were plated in triplicate. Experiments were performed three times. Two slides containing three sections each were subject to immunostaining. Mean number of spheroids analyzed per group±s.e.m.: pHAGE, 275±58; ALDH-OE, 219±38. Statistical analysis was performed using one-way ANOVA with Bonferroni post-hoc comparison. *P<0.05; n.s, not significant. Mean±s.e.m. is shown.

We modulated ALDH1A1 expression in CCR2-overexpressing and CCR2-deficient cells to determine its contribution to CCL2/CCR2-mediated growth and invasion. As CCL2 treatment enhanced ALDH1A1 expression and activity in CCR2-H SUM225 cells, ALDH1A1 was knocked down in these cells through stable shRNA expression. In CCR2-H cell lines expressing two different targeting shRNAs reduced ALDH1A1 expression to 36% (ALDH-KD#1) and to 26.8% (ALDH-KD#5) relative to levels detected in control cells (Fig. 6A). ALDH1A1 knockdown did not significantly affect HTRA2 expression. ALDH1-KD#1 did not show changes in spheroid size or invasiveness. ALDH-KD#5 cells showed a 20% decrease in spheroid size associated with decreased cell proliferation, but not invasion or changes in E-cadherin or TWIST1 expression in 3D cultures (Fig. 6B–D, Figs S6 and S7A,B). These data indicate an association between the level of ALDH1A1 knockdown to effects on cell proliferation in CCR2-overexpressing SUM225 cells. We examined the role of ALDH1A1 in DCIS.com cells. CCR2-deficient DCIS.com cells inhibited CCL2-induced ALDH1A1 expression. Therefore, ALDH1A1 was stably overexpressed in CCR2-KO DCIS.com cells through lentiviral transduction (Fig. 6E). ALDH1A1 overexpression did not significantly affect HTRA2 expression. Compared to DCIS.com CCR2-KO cells transduced with pHAGE control lentivirus, ALDH1A1 overexpression increased spheroid size in 3D cultures, associated with increased cell proliferation but not cellular invasion, or changes to E-cadherin or TWIST1 expression (Fig. 6F–H, Fig. S7C,D). In summary, these data indicate that ALDH1A1 is important for CCL2/CCR2-mediated breast cancer cell growth, but not invasion.

**Fig. 7.**
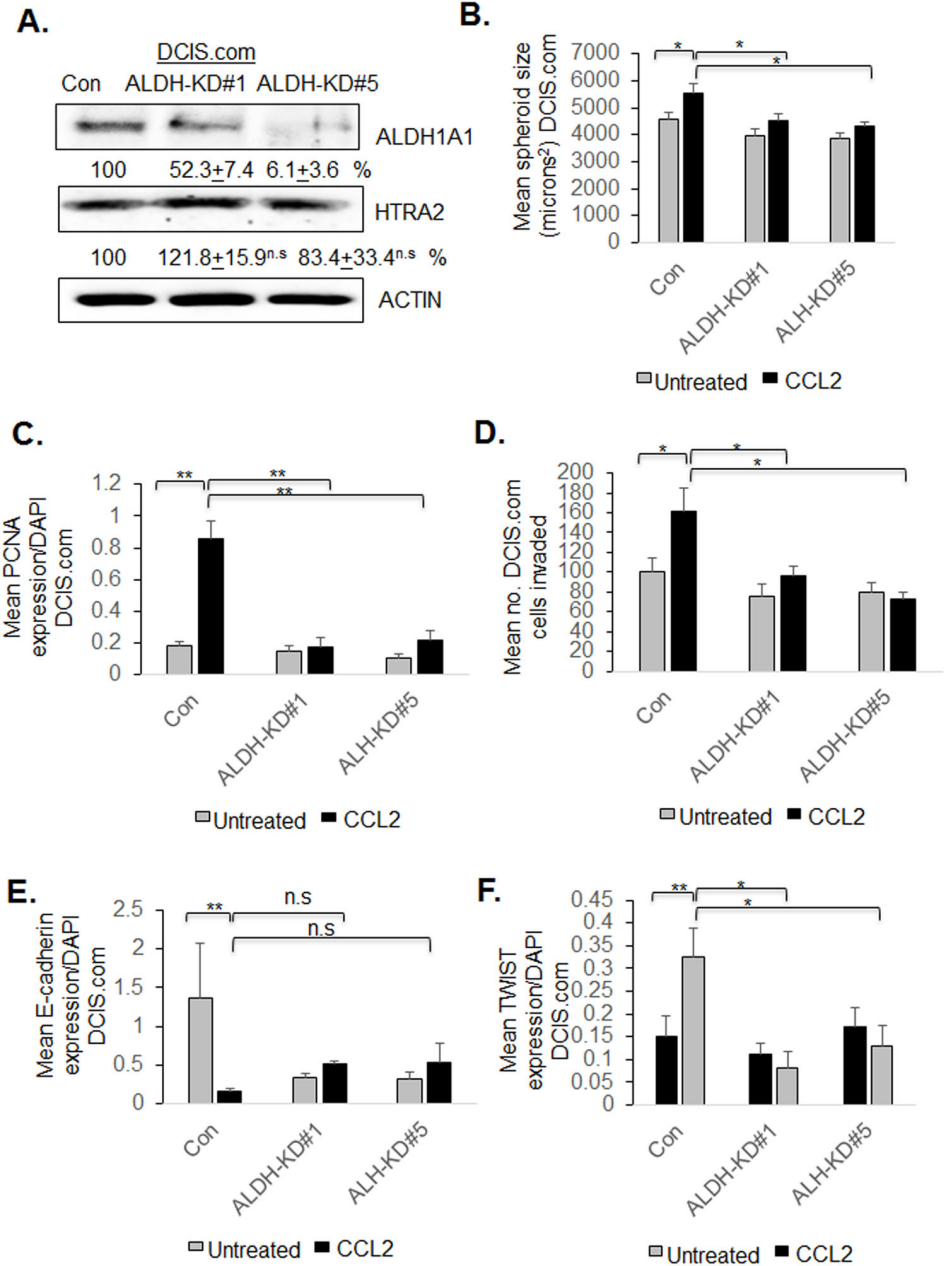
**ALDH1A1 knockdown inhibits DCIS.com breast cancer cell growth and invasion.** (A–F) DCIS.com cells expressing control shRNAs (Con) or shRNAs to ALDH1A1 (ALDH-KD#1, ALDH-KD#5) were treated with/without 100 ng/ml CCL2 and analyzed for (A) ALDH1A1 and HTRA2 expression by immunoblot, (B) spheroid size in 3D cultures, (C) PCNA expression, (D) transwell invasion, (E) E-cadherin or (F) TWIST1 expression. Mean number of spheroids analyzed per group±s.e.m.: Con, 163+9; Con+CCL2, 165+7; ALDH-KD#1 152+12; ALDH-KD#1+CCL2, 168+11; ALDH-KD#5, 174+10; ALDH-KD#5+CCL2, 162+13. Protein levels in immunoblots were measured by densitometry. Expression levels were normalized to control group. Representative blots of three experiments are shown. Spheroid size and invasion index were normalized to spheroid number. Experimental groups were plated in triplicate. Experiments were performed three times. Two slides containing three sections each were subject to immunostaining. Statistical analysis was performed using one-way ANOVA with Bonferroni post-hoc comparison. *P<0.05, **P<0.01; n.s, not significant. Mean±s.e.m. is shown.

To examine how ALDH1A1 functioned in a breast cancer cell line with high levels of endogenous CCR2, we knocked down ALDH1A1 by stable shRNA expression in parental DCIS.com cells. In ALDH1A1-KD#1 cells, ALDH1A1 was knocked down to 52.3% relative to control cells. In ALDH1A1-KD#5 cells, ALDH11A1 was decreased to 6% relative to control cells. (Fig. 7A). ALDH1A1 knockdown did not affect HTRA2 expression. ALDH1-KD#1 and ALDH1-KD#5 showed a significant decrease in CCL2-induced DCIS.com spheroid growth in 3D cultures, associated with decreased PCNA expression (Fig. 7B,C). ALDH1A1 knockdown did not affect basal level spheroid growth or PCNA expression. By transwell assay, ALDH1-KD#5 did not affect basal level DCIS.com invasion, but inhibited CCL2-induced invasion (Fig. 7D). ALDH1A1 knockdown resulted in a small but not statistically significant increase in E-cadherin expression in CCL2-treated cells (Fig. 7E) and significantly inhibited CCL2-induced TWIST1 expression (Fig. 7F). In summary, these data indicate that in parental DCIS.com cells, ALDH1A1 regulates CCL2-induced growth and invasion.

### HTRA2 negatively regulates CCR2-mediated breast cancer cell growth and invasion in a CCL2/CCR2 context-dependent manner

**Fig. 8.**
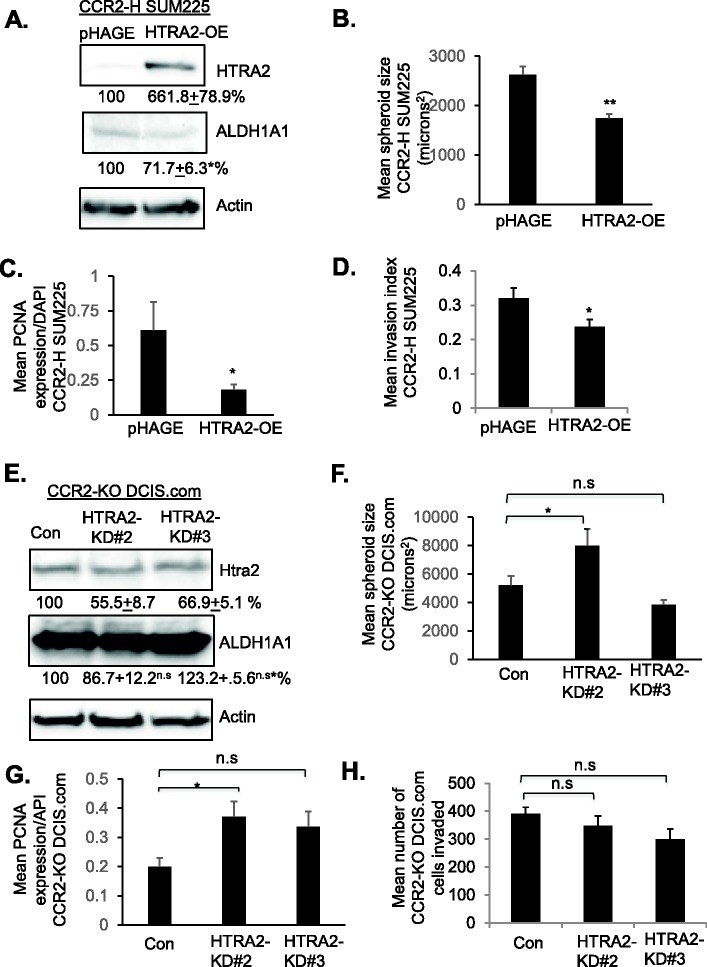
**HTRA2 overexpression or** **knockdown affects CCR2-H SUM225 and CCR2-KO DCIS.com breast cancer cell growth and invasion.** (A–D) CCR2-H SUM225 cells expressing vehicle control (pHAGE) or HTRA2 (HTRA2-OE) were analyzed for HTRA2 expression by immunoblot (A), spheroid size in 3D cultures (B), PCNA expression (C) or invasion index (D). Mean number of spheroids analyzed per group±s.e.m.: pHAGE, 269±63; HTRA2-OE, 227±28. (E–H) CCR2-KO DCIS.com cells were transduced with lentivirus expressing Con or HTRA2 shRNAs (HTRA2-KD#2, HTRA2-KD#3) and analyzed for HTRA2 expression by immunoblot (E), spheroid size in Matrigel: Collagen (F), PCNA expression (G) or transwell invasion (H). Spheroid size and invasion index were normalized to spheroid number. Experimental groups were plated in triplicate. Experiments were repeated three times. Two slides containing three sections each were subject to immunostaining. Mean number of spheroids analyzed/group±s.e.m.: Con, 196±80; HTRA2-KD#2, 219±99; HTRA2-KD#2, 161±40. Statistical analysis was performed using two-tailed *t*-test (A–D) or one-way ANOVA with Bonferroni post-hoc comparison (E–H). *P<0.05, **P<0.01, ***P<0.001; n.s, not significant. Mean±s.e.m. is shown.

We then modulated HTRA2 expression in CCR2-overexpressing and CCR2-deficient cells to determine its contribution to CCL2/CCR2-mediated growth and invasion. As CCR2 overexpression in SUM225 cells decreased HTRA2 expression, HTRA2 expression was rescued through lentiviral transduction of CCR2-H cells. Interestingly, HTRA2 rescue significantly decreased ALDH1A1 expression by 28.3% in CCR2-H SUM225 cells (Fig. 8A). HTRA2 overexpression inhibited spheroid size, associated with decreased cell proliferation and invasion of CCR2-H cells, associated with decreased TWIST1 and increased E-cadherin expression (Fig. 8B–D, Figs S8 and S9A-B). As a complementary approach, HTRA2 expression was knocked down in CCR2-KO DCIS.com cells through shRNA expression. Relative to control cells, HTRA2 expression was decreased to 55.5% in HTRA2-KD#2 cells, and decreased to 66.9% in HTRA2-KD#3 cells (Fig. 8E). ALDH1A1 expression was not significantly affected with HTRA2 knockdown in CCR2-KO DCIS.com cells. CCR2-KO cells expressing HTRA2-KD#2 but not HTRA2-KD#3 shRNAs showed a significant increase in spheroid size associated with increased cell proliferation (Fig. 8F,G). HTRA2-KD#2 and HTRA2-KD#3 did not show significant changes in cellular invasion, E-cadherin or TWIST1 expression (Fig. 8H, Fig. S9C,D). In summary, these data indicate that HTRA2 suppresses CCR2-mediated cell proliferation in SUM225 and DCIS.com cells, and suppresses invasion of CCR2-overexpressing SUM225 cells, but not DCIS.com cells.

**Fig. 9.**
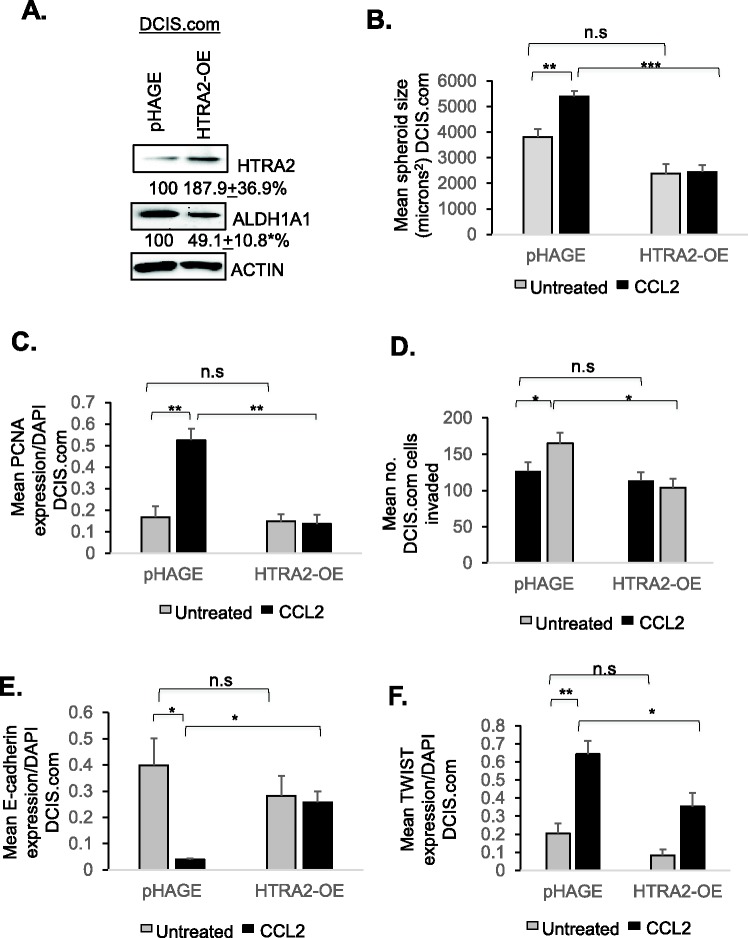
**HTRA2 overexpression inhibits** **DCIS.com breast cancer cell growth and invasion.** (A–F) DCIS.com cells expressing pHAGE control or HTRA2 were analyzed for (A) ALDH1A1 and HTRA2 expression by immunoblot, (B) spheroid size in 3D cultures, (C) PCNA expression, (D) transwell invasion, (E) E-cadherin or (F) TWIST1 expression. Mean number of spheroids analyzed per group±s.e.m.: pHAGE, 325±25; pHAGE±CCL2, 358±33; HTRA2-OE, 343±17; HTRA2-OE±CCL2, 316±44. Protein levels in immunoblots were measured by densitometry. Expression levels were normalized to control group. Representative blots of three experiments are shown. Spheroid size and invasion index were normalized to spheroid number. Experimental groups were plated in triplicate. Experiments were performed three times. Two slides containing three sections each were subject to immunostaining. Statistical analysis was performed using two-tailed *t*-test (A) or one-way ANOVA with Bonferroni post-hoc comparison (B–F). *P<0.05, **P<0.01, ***P<0.001; n.s, not significant. Mean±s.e.m. is shown.

To examine how HTRA2 functioned in a breast cancer cell line with high levels of endogenous CCR2, we overexpressed HTRA2 in parental DCIS.com cells. DCIS.com cells showed robust HTRA2 overexpression, which did not significantly affect ALDH1A1 expression (Fig. 9A). HTRA2 overexpression inhibited basal level and CCL2-induced DCIS.com spheroid growth in 3D cultures, associated with decreased PCNA expression (Fig. 9B,C). By transwell assay, HTRA2 overexpression did not affect basal level DCIS.com invasion, but did inhibit CCL2-induced invasion (Fig. 9D), associated with increased E-cadherin expression and decreased TWIST1 expression (Fig. 9E,F) In summary, these data indicate that in parental DCIS.com cells, HTRA2 negatively regulates CCL2-induced growth and invasion.

### Associations between p42/44MAPK and SMAD3 activity with CCL2-mediated ALDH1A1 and HTRA2 expression in DCIS.com and CCR2-overexpressing SUM225 cells

**Fig. 10.**
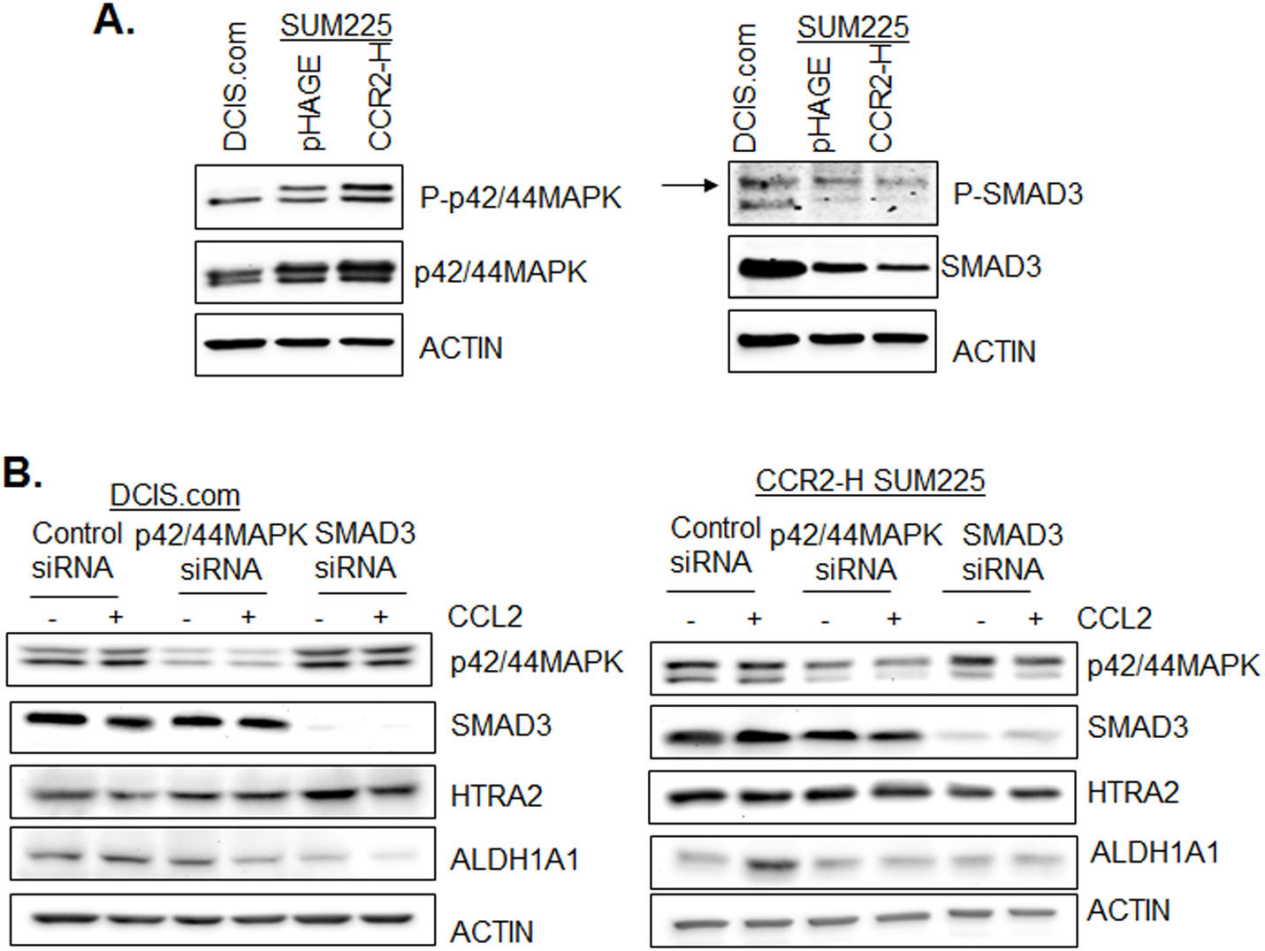
**Effect of p42/44MAPK and SMAD3 knockdown on CCL2/CCR2-mediated ALDH1A1 and HTRA2 expression.** (A) DCIS.com, pHAGE or CCR2-H SUM225 cell lines were analyzed for expression of the indicated proteins by immunoblot. Arrow points to SMAD3 protein approximately 50 kda in size. (B) DCIS.com or CCR2-H SUM225 cells were transfected with control siRNAs or siRNAs to p42/44MAPK or SMAD3, stimulated with CCL2 for 24 h and analyzed for expression of the indicated proteins by immunoblot.

In previous studies, we demonstrated an important role for p42/44MAPK and SMAD3 in CCL2/CCR2 breast cancer cell motility (Fang et al., 2012). Compared to pHAGE vehicle control and parental DCIS.com cells, CCR2-H SUM225 cells showed higher levels of phospho-p42/44MAPK and total p42/44MAPK expression. Parental DCIS.com cells showed higher levels of phospho-SMAD3 and SMAD3 protein compared to pHAGE or CCR2-SUM225 cells (Fig. 10A). To determine the relevance of p42/44MAPK and SMAD3 to ALDH1A1 and HTRA2 expression, we knocked down expression of p42/44MAPK or SMAD3 by siRNA transfection in DCIS.com and CCR2-H SUM225 cells, treated with or without CCL2 and examined for ALDH1A1 and HTRA2 expression by immunoblot. In DCIS.com cells, p42/44MAPK and SMAD3 knockdown inhibited ALDH1A1 expression in CCL2-treated and untreated cells, and increased HTRA2 expression in CCL2-treated and untreated cells. In CCR2-H SUM225 cells, p42/44MAPK and SMAD3 knockdown did not affect ALDH1A1 expression in untreated cells, but inhibited CCL2 induced ALDH1A1 expression. p42/44MAPK and SMAD3 knockdown did not visibly affect HTRA2 expression in untreated or CCL2-treated cells (Fig. 10B). These data indicate that p42/44MAPK and SMAD3 signaling pathways positive regulate CCL2-mediated ALDH1A1 and negatively regulate HTRA2 expression in DCIS.com cells. In CCR2-H SUM225 cells, p42/44MAPK and SMAD3 positively regulate ALDH1A1 expression, and do not affect HTRA2 expression. In summary, these data indicate differences in intracellular signaling in DCIS.com and CCR2-overexpressing SUM225 cells that influence ALDH1A1 and HTRA2 expression.

## DISCUSSION

Recent studies have shown that CCL2/CCR2 chemokine signaling in carcinoma cells enhances breast cancer growth and invasion (Brummer et al., 2018). However, the mechanisms through which CCL2/CCR2 signaling promotes cancer progression are poorly understood. Using 3D cell culture and transwell invasion systems, we report that CCL2/CCR2 signaling regulates breast cancer cell growth and invasion in part by enhancing ALDH1A1 expression and suppressing HTRA2 expression. However, the contributions of ALDH1A1 and HTRA2 to breast cancer cell growth and invasion may depend on a complex set of factors involving differences in CCL2/CCR2 signaling among breast cancer cell lines.

CCL2/CCR2-mediated breast cancer progression was associated with increased growth, invasion and decreased tumor cell apoptosis (Brummer et al., 2018). Here, we found that CCL2/CCR2 signaling in DCIS.com and SUM225 cells in 3D cultures enhanced proliferation and invasion, but had no effect on apoptosis. It is possible that differences in 3D cultures and tissues may account for differences towards apoptosis. For one, 3D cultures lacked stromal components that may affect breast cancer cell survival. CCR2 overexpression in breast lesions was associated with accumulation of fibroblasts (Brummer et al., 2018). In addition to CCL2, fibroblasts secrete other pro-survival factors such as HGF and TGF-β (Soriano et al., 1998). These factors act directly on breast cancer cells to inhibit programmed cell death (Zhang et al., 2017). In addition, TGF-β promote tumor cell survival by suppressing CD8+ T cell function, differentiate pro-inflammatory T cell populations and enhance polarization of myeloid cells to a wound-healing phenotype (Dahmani and Delisle, 2018; Fridlender et al., 2009; Gratchev, 2017). While there are some limitations to 3D culture systems, our studies indicate that some tumor phenotypes such as cell growth and invasion are reproduced *in vitro*.

While CCL2 is expressed in carcinoma cells, studies have indicated that CCL2 expression in the stroma is a more significant prognostic indicator in breast cancer (Fujimoto et al., 2009; Yao et al., 2016b). Furthermore, CCL2 derived from fibroblasts is important for enhancing breast tumor growth, invasion and metastasis (Brummer et al., 2018; Hembruff et al., 2010). These data suggest that paracrine CCL2 signaling to breast cancer cells is an important mechanism for CCR2-mediated breast cancer progression. Here, CCL2 stimulation of CCR2 overexpressing cells enhanced growth and invasion more than CCR2 overexpression alone, further supporting an important role for paracrine CCL2/CCR2 signaling to breast cancer cells. Interestingly, CCR2 overexpression alone in SUM225 cells enhanced invasion associated with increased CCL2 expression and p42/44MAPK signaling, indicating that breast cancer cell invasion may also be regulated in part by autocrine CCL2/CCR2 signaling mechanisms, characterized in part by p42/44MAPK activity. In addition to p42/44MAPK, CCL2/CCR2 activates multiple signaling pathways in breast cancer cells including: PKC, Rho and SRC to regulate growth and motility (Fang et al., 2012). These pathways are also important in breast cancer cell invasion (Gautam et al., 2018; Kim et al., 2017; Matsuura et al., 2010; Shi et al., 2017). It would be of interest in the future to distinguish which pathways are mediated by paracrine versus autocrine CCL2/CCR2 signaling in breast cancer cells to regulate cell growth and invasion.

In previous studies of animal models, we showed that CCR2 expression positively regulated breast carcinoma growth and invasion (Brummer et al., 2018; Yao et al., 2019). Here, we observed that CCR2 overexpression and CCR2 deficiency in breast cancer cells resulted in some complementary phenotypes. CCL2 treatment of CCR2-overexpressing SUM225 cells enhanced spheroid growth and cell invasion, associated with increased ALDH1A1 and decreased HTRA2 expression. Conversely, CCR2 knockdown or knockout in DCIS.com cells inhibited CCL2-induced: spheroid growth, cell invasion and ALDH1A1 expression, and prevented CCL2 suppression of HTRA2 expression. Changes in cellular invasion were accompanied by consistent changes in E-cadherin and TWIST1 expression. However, some conflicting phenotypes were observed. CCR2 overexpression alone in SUM225 cells was sufficient to enhance invasion. Yet, CCR2 deficiency in DCIS.com cells alone did not show differences in invasion. These studies suggest that other oncogenic pathways may be involved in malignancy of DCIS.com cells. Pathways such as TGF-β or IL-8 regulate EMT and invasion (Bhowmick et al., 2001; Zhang et al., 2016), and may compensate for loss of CCR2 expression to sustain invasion in more tumorigenic cell lines, such as DCIS.com. It is also possible that CCR2 may coordinate growth and invasion in DCIS.com cells with other oncogenes that are not present in SUM225 cells. G Protein Coupled Receptors crosstalk with receptor tyrosine kinases such as EGFR (Wang, 2016), which are overexpressed in breast cancers (Sainsbury et al., 1987; Toi et al., 1991). In future studies, it would be of interest to examine how CCR2 coordinates breast tumor growth and invasion with other oncogenic factors.

Here, we found that CCL2 treatment combined with CCR2 overexpression significantly enhanced ALDH1A1 expression and activity in CCR2-H SUM225 and DCIS.com cells. ALDH1A1 is a highly conserved enzyme dehydrogenase in alcohol metabolism, and functions to convert acetaldehyde to acetate (Koppaka et al., 2012; Smith et al., 2015), and converts all trans- and 9 cis- retinaldehydes to retinoic acid (RA) (Fan et al., 2003; Paterson et al., 2013) during melanogenesis and retina development. ALDH1A1 expression and activity have been implicated in tumor cell proliferation, cancer stem cell renewal, invasion and drug resistance (Charafe-Jauffret et al., 2010; Ginestier et al., 2007; Rodriguez-Torres and Allan, 2016; Xu et al., 2015). We noted that WT control cells showed greater ALDH1A1 expression and activity with CCL2 treatment compared to control shRNA expressing cells. The differences in ALDH1A1 and Aldered activity could be due to inherent genetic or epigenetic differences when shRNA expressing cells and CRISP/R knockout cells were generated. CCR2 shRNAs were expressed in a heterogenous pool of DCIS.com cells. CCR2-KO was generated through clonal selection during the CRISPR process (Brummer et al., 2018). Despite the differences in how these cells were generated, CCR2 shRNA knockdown and CRISPR knockout both led to a reduction in breast cancer survival and invasion in animal models (Brummer et al., 2018). Here, CCL2 treatment enhanced ALDH1A1 expression and Aldered activity in both control cell lines, which were inhibited by CCR2 shRNA or CRISP/R knockout.

Altering ALDH1A1 expression in CCR2-H SUM225, CCR2-KO and parental DCIS.com cells led to changes in spheroid size and PCNA expression, indicating that an important role for ALDH1A1 in breast cancer cell growth regardless of cell line or CCR2 status. The exact molecular mechanisms through which ALDH1A1 selectively regulates growth in breast cancer cells is unclear, but could involve FOXM1 and Notch signaling. ALDH1A1 activates FOXM1 and Notch1 to promote ovarian tumorigenesis and stemness (Young et al., 2015). FOXM1 and Notch1 regulate expression of cell cycle and metabolic genes in cancer (Sanchez-Martin and Ferrando, 2017; Wierstra and Alves, 2007; Yao et al., 2018). Alternatively, ALDH1A1 conversion of retinaldehydes to RA could serve to enhance breast cancer proliferation. RA exerts its biological effects by activating nuclear receptors PPAR β/δ and facilitating RA Receptor (RAR) and Retinoid X Receptor (RXR) complexes to response elements for gene transcription (Schug et al., 2007; Singh et al., 2015). RA activity can promote growth of pancreatic cancers or inhibit colon cancer growth (Balasubramanian et al., 2004; Mollersen et al., 2004). RAR and RXR have been implicated in pro-apoptotic and suppression of cell growth (Schug et al., 2007). Yet, RA can also activate pathways involved in cell proliferation and stem cell activity including p42/44MAPK (Yen et al., 1998), PKC (Wu et al., 2017) and p38MAPK (Alsayed et al., 2001). Alternatively, ALDH1A1 may have other substrates that are involved in breast cancer cell growth, which could be identified through a broad high-throughput substrate screen. In summary, it is possible that ALDH1A1 expression and activity in DCIS.com and SUM225 CCR2-overexpressing breast cancer cells may facilitate cell growth through metabolites that activate oncogenic pathways.

Interestingly, we noticed differences in the effects of modulating ALDH1A1 expression in parental DCIS.com, CCR2-H SUM225 and CCR2-KO DCIS.com cell invasion. In CCR2-KO DCIS.com cells, ALDH1A1 overexpression did not rescue invasion. This may be because these cells lacked other components required for cellular invasion. For example, CCR2 deficiency in DCIS.com cells inhibited TWIST1 expression. TWIST1 expression regulates expression of genes associated with cellular adhesion, cell motility and actin cytoskeletal reorganization (Mikheeva et al., 2010). It is possible that CCR2-KO of DCIS.com cells inhibited one or more of these critical factors, and therefore, ALDH1A1 overexpression by itself was not sufficient to restore invasion. In CCR2-H SUM225 cells, ALDH1A1 knockdown did not affect invasion. However, in parental DCIS.com cells, ALDH1A1 knockdown inhibited CCL2 induced cellular invasion. The contributions of ALDH1A1 to invasion of CCR2-H SUM225 versus parental DCIS.com cells may due to differences in intracellular signaling. CCR2-H SUM225 showed increased p42/44MAPK while DCIS.com cells showed higher levels of SMAD3 signaling, which could regulate invasion of genes associated with invasion or modulate changes in actin cytoskeletal reorganization that works with ALDH1A1 to regulate invasion. In addition, DCIS.com cells showed increased RAS overexpression (Miller et al., 2000). In pancreatic cancer, RAS-mediated invasion and motility was associated with increased ALDH1A1 expression (Rachagani et al., 2011). In DCIS.com cells, RAS may drive ALDH1A1 expression, which activate NOTCH1 to enhance EMT and invasion (Shao et al., 2015; Young et al., 2015). It is possible that the differences in RAS expression may contribute to how ALDH1A1 functions in CCR2-H SUM225 cells versus parental DCIS.com cells.

Our studies indicate that HTRA2 is important in CCL2/CCR2-mediated breast cancer cell growth and invasion, but not apoptosis. HTRA2 is best known for its role as an apoptotic inducer through binding and cleaving inhibitors of apoptosis proteins (Guo et al., 2016; Kilbride and Prehn, 2013). A few studies have indicated that HTRA2 may have other cellular functions. Mice exhibiting knockout of HTRA2 (Omi) do not show changes in cell death in tissues (Martins et al., 2004). Another study has shown that HTRA2 suppresses cell proliferation through binding to WARTS (WTS)/large tumor-suppressor 1 mitotic kinase (Kuninaka et al., 2007). HTRA2 cleaves STAT3 protein to modulate expression of cytokines that regulate T cell proliferation (Lee et al., 2016). These studies indicate that HTRA2 cleaves multiple substrates, including proteins that directly regulate the cell cycle, or indirectly, through modulating activity of transcription factors involved in cell proliferation. HTRA2 may function downstream of CCL2/CCR2 to regulate cell proliferation and invasion through similar mechanisms.

Modulating HTRA2 expression in CCR2-H SUM225, CCR2-KO and parental DCIS.com cells led to changes in spheroid size and PCNA expression, indicating that HTRA2 exerts important effects on breast cancer cell growth regardless of cell line or CCR2 status. Yet, modulating HTRA2 expression affected breast cancer cell invasion differently among the three cell lines. HTRA2 overexpression in CCR2-H SUM225 cells decreased cell invasion, indicating that CCR2-mediated SUM225 cellular invasion is directly associated with HTRA2 expression. In CCR2 deficient DCIS.com cells, HTRA2 knockdown did not restore cellular invasion, suggesting that CCR2 deficiency inhibits expression or activity of other components required for cellular invasion such as TWIST1. HTRA2 overexpression in parental DCIS.com cells alone led to a small decrease in invasion and significantly inhibited CCL2-induced invasion, suggesting that CCL2-stimulated invasion is dependent on suppression of HTRA2 expression. In mouse embryonic fibroblasts and NIH3T3 deficient for p53, RAS transforms cells and promotes cellular invasion by preventing HTRA2-mediated cleavage of beta actin, enabling phosphorylating p130Cas to promote formation of integrin adhesion complexes and lamellipodia (Yamauchi et al., 2014). In parental DCIS.com cells, a similar mechanism may occur in which CCL2-mediated RAS activity may prevent HTRA2-mediated cleavage of beta actin. In addition, RAS activates signaling pathways including p42/44MAPK, which negatively regulated HTRA2 expression in DCIS.com cells. RAS also regulates PI3kinase, AKT and PKC pathways, all of which modulate activity of transcription factors (Bos, 2018), and may also suppress HTRA2 expression in DCIS.com cells.

In CCR2-H SUM225 cells, HTRA2 overexpression decreased ALDH1A1 expression, while HTRA2 knockdown in CCR2-KO DCIS.com cells and HTRA2 overexpression in parental cells did not affect ALDH1A1 expression. In SUM225 cells, CCR2 overexpression may enhance mechanisms that couple HTRA2 to ALDH1A1 expression. Proteomics profiling of Jurkat T cells identified HTRA2 substrates including: ALDH isoforms as substrates (Vande Walle et al., 2007). Alternatively, HTRA2 cleaves co-factors such as E2F1 (Ma et al., 2015) that interact with NF-Y to regulate ALDH1A1 promoter activity (van Ginkel et al., 1997; Yanagawa et al., 1995). It is possible that CCR2 overexpression decreased HTRA2 expression to stabilize ALDH1A1 protein levels or gene expression. In contrast, for CCR2-KO and parental DCIS.com cells, HTRA2 and ALDH1A1 could function independently of each other, or multiple pathways regulate HTRA2 and ALDH1A1 expression. As such, modulating HTRA2 or ALDH1A1 expression would not affect expression of the other gene.

Interestingly, CCR2 overexpression in SUM225 cells resulted in some differences in p42/44MAPK and SMAD3 expression and activity compared to DCIS.com cells. p42/44MAPK and SMAD3 positively regulated ALDH1A1 expression and negatively regulated HTRA2 expression in CCL2-treated DCIS.com cells. SMAD3 and p42/44MAPK positively regulated CCL2-induced ALDH1A1 expression and did not affect HTRA2 expression in CCR2-H SUM225 cells. These differences in intracellular signaling, and regulation of ALDH1A1 and HTRA2 expression could dictate how CCL2/CCR2 regulates cell growth, invasion in CCR2-H SUM225 cells and DCIS.com cells. The mechanisms responsible for these differences in intracellular signaling are unclear, but could in part relate to differences in mechanisms that control p42/44MAPK and SMAD3 expression and activity in DCIS.com cells and SUM225 cells. p42/44MAPK is regulated by a number of negative and positive feedback mechanisms. Ras GAP protein regulates Ras activation of Raf, MEK and subsequently p42/44MAPK (Hennig et al., 2016). One mechanism is Kinase Suppressor of Ras proteins, which act as a scaffold for p42/44MAPK pathway and regulate localization of p42/44MAPK to Raf-1 (Frodyma et al., 2017). SMAD3 gene expression is regulated by the TGF-β pathway (Zhao and Geverd, 2002). SMAD binding proteins such as SnoN, SKI and Protein Kinase B/AKT regulate SMAD3 activity and sequestration to the cyotoplasm (Conery et al., 2004). It would be interest in the future to understand how CCR2 regulates growth, invasion, ALDH1A1 and HTRA2 expression and activity in different cell lines through examination of other signal transduction pathways.

In summary, this study demonstrates that CCL2/CCR2 chemokine signaling in breast cancer cells is an important regulatory mechanism for cell growth and invasion. CCL2 and CCR2 are currently therapeutic targets of interest for anti-cancer treatment and inflammatory diseases (Bertrand and Tardif, 2017; Lim et al., 2016). Patients diagnosed with invasive or metastatic breast cancer have a more unfavorable prognosis than patients with non-invasive disease (DeSantis et al., 2017). By understanding the molecular and cellular mechanisms of CCL2/CCR2 signaling facilitating growth and invasion of cancer cells, we may better understand the potential effects of CCL2 or CCR2 inhibitors on tumor progression, and design improved strategies for treatment of invasive breast cancer.

## MATERIALS AND METHODS

### Cell culture

DCIS.com and SUM225 breast cancer cells were kindly provided by Dr Fariba Behbod (University of Kansas Medical Center). SUM225 cells were maintained in Ham's F-12 medium containing: 10% Fetal Bovine Serum (FBS), 0.1% amphotericin and 0.1% penicillin-streptomycin. DCIS.com cells were maintained in DMEM with 10% FBS/0.1% amphotericin/0.1% penicillin-streptomycin. Cells were analyzed for mycoplasma after every freeze/thaw using the MycoAlert™ Mycoplasma Detection Kit (Lonza, cat. no. LT07-318). Cells were maintained no longer than 3 months at a time.

### Lentiviral transduction

The following plasmids were used to create cell lines: GIPZ ALDH1a1 shRNA, glycerol set (RHS4531-EG216): V2LHS_112035, V2LHS_112037, V2LHS_112039, V2LHS_265598, V3LHS_398453, V3LHS_398455. GIPZ HTRA2 shRNA, glycerol set (RHS4531-EG27429): V3LHS_315862, V3LHS_315863, V3LHS_315864, V3LHS_3155866.

MGC Human HTRA2 sequence-verified cDNA (3508944), MGC Human ALDH1a1 sequence verified-cDNA (2988388). To generate lentivirus, 293T cells were transduced 6 μg psPAX2, 3 μg pMD.2G and 10 μg of the plasmid of interest. The media was harvested twice a day for 3 days, and concentrated in 8.5% PEG 6000 and 0.4 M NaCl. Parental DCIS.com, CCR2-KO DCIS.com and CCR2-H SUM225 were transduced with lentivirus for 24 h before the media was replaced.

### siRNA transfection

40,000 cells were seeded into 24-well plates for 24 h. 8 pmol of siRNAs to control (Ambion, cat. no. AM4613), SMAD3 (Santa Cruz Biotechnology, cat. no. sc-38376), p42MAPK (Santa Cruz Biotechnology cat. no. sc-29307) mixed with p44MAPK (Santa Cruz Biotechnology, cat. no. 35335) were complexed at a 1:10 ratio to lipofectamine 2000 (Invitrogen cat. no. 11668027) and transfected into cells for 24 h in DMEM. The media was replaced with DMEM/10% FBS for 24 h prior to CCL2 stimulation.

### 3D cell culture

Cells were cultured in Collagen: Matrigel, using procedures adapted from previous studies (Berens et al., 2015). Rat tail collagen (3.5 mg/ml) (Corning, cat. no. 354236) was mixed at a 4:1 ratio with setting solution comprised of: 1× EBSS, 75 mM NaOH and 290 mM NaHCO_3_. The collagen solution was then diluted 1:1 with Growth Factor Reduced Matrigel (BD Pharmingen, cat. no. 354230). 96-well plates were coated with 40 µl/well of the Matrigel: Collagen solution for 30 min at 37°C. Breast cancer cells (2500) were re-suspended in 200 µl growth medium containing 2.5% Matrigel and seeded in each well for 24 h. Cells were treated with or without recombinant human CCL2 (Peprotech, cat. no. 300-04) at 60 ng/ml or 100 ng/ml. Cells were cultured for up to 10 days, with the medium changed at day 4 and 8. Images were captured every 2 days using the EVOS FL Auto Imaging System (Invitrogen) at 10× magnification, four fields per well.

### Immunofluorescence

Immunofluorescence staining of 3D cultures was adapted from previous studies (Artym and Matsumoto, 2010). 3D culture spheroids were fixed in 10% neutral buffered formalin overnight at 4°C. Samples were removed from 96-well plates and embedded in 4% agarose plugs. Samples were dehydrated in 70, 90, 100% ethanols for 1 h each, and embedded in paraffin blocks. 5 µm sections were de-waxed. To detect E-cadherin, sections were permeabilized in PBS/0.1%TritonX-100 for 10 min, blocked with PBS/3% FBS, incubated at 1:100 with E-cadherin antibodies (BD Pharmingen, cat. no. 610181) overnight. Sections were then incubated with donkey anti-rabbit-AlexaFluor-647 (Invitrogen, cat. no. A-31573) at a 1:250 dilution for 2 h. For all other antigens, de-waxed sections were subject to antigen retrieval by heating in 10 mM Sodium Citrate, pH 6.0 for 2 min at low pressure in a pressure cooker. Sections were permeabilized in PBS containing 10% Methanol for 10 min. Slides were blocked in PBS/3% FBS, and incubated overnight with antibodies (1:100) to: Cleaved Caspase-3 (Cell Signaling Technology, cat. no. 9661S), or TWIST1 (Santa Cruz Biotechnology, cat. no. sc.81417), PCNA (BioLegend, cat. no. 307902), HTRA2 (Cell Signaling Technology, cat. no. 2176S) or ALDH1A1 (RnD system, cat. no. MAB5869). Slides were washed in PBS. PCNA was detected with donkey anti-rabbit-AlexaFluor-647. Cleaved caspase-3 and HTRA2 were detected with biotinylated rabbit antibodies (Vector Laboratories, cat. no. BA1000), followed by AlexaFlour-568 conjugated streptavidin (Vector Laboratories, cat. no. PK-4000). ALDH1A1 and TWIST1 was detected with biotinylated mouse antibodies (Vector Laboratories, cat. no. BA9200), followed by Alexa-Fluor-568 conjugated to streptavidin. Slides were counterstained with DAPI, and mounted with 1:1 PBS: glycerol. Six random fields per sample were captured at 10× magnification using the EVOS FL Auto Imaging System. Antibody specificity was controlled through shRNA knockdown and overexpression analysis by immunoblot, peptide competition assay and omission of primary antibody staining, Expression was quantified by ImageJ, using approaches described previously (Cheng et al., 2008). Expression was normalized to DAPI.

### Aldered assay

9 days after culture in Matrigel: Collagen, cells were subject to Aldered ALDH detection assay (Millipore, cat. no. SCR150). Aldered-588 reagent was prepared in assay buffer containing Verapamil to prevent efflux, according to manufacturer's instructions. 5 µl of Aldered solution was added to each with or without DEAB for 60 min at 37°C. Cells were counterstained with DAPI. Fpur images per field at 10× magnification were captured using the FL Auto EVOS Imaging system. Expression was quantified by ImageJ, using approaches described previously (Cheng et al., 2008). Expression was normalized to DAPI.

### Quantitation of spheroid size and invasion

Images were opened in ImageJ. The total size was measured by outlining the size of each cell cluster, adjusting with threshold analysis and analyzed for total area. Pixel units were converted to microns (1 pixel^2^=1.125 µm^2^). The invasion index was calculated as described in previous studies (Cisneros Castillo et al., 2016). The main spheroid body (spheroid minus invasive protrusions) was outlined, and measured for area. The invasion index was calculated by dividing total area by main body area of each spheroid and subtracting from 1.

### Ca-TAT transfection

TAT peptides [sequence N to C: (+)H-RKKRRQRRR-NH2(+)] were synthesized to purity >95% by Biomatik (Cambridge, Canada). Ca-TAT/siRNA complexes were prepared at N/P 5 ratio using a formula adapted from previous studies (Baoum et al., 2012). 1.41 µg TAT peptides were mixed with 6 µg pHAGE–CMV-MCS-IRES-zsgreen control plasmids or pHAGE plasmid carrying full length CCR2 cDNA (Brummer et al., 2018) in 45 µl sterile de-ionized water containing 75 mM CaCl_2_. The solution was pipetted 20 times and incubated on ice for 20 min. These complexes were added to 3D cultures 2 days after cells were seeded.

### Immunoblot

Cells were lysed in buffer containing: 10 mM Tris-HCl, pH 8.0, 0.1 mM EDTA, 0.1% sodium deoxycholate, 0.1% SDS and 140 mM NaCl with protease and phosphatase inhibitors (Sigma-Aldrich, cat. no. P8340). 25 μg of protein were resolved by 10% SDS-PAGE, transferred to nitrocellulose membranes and probed with antibodies diluted 1:1000 to: ALDH1A1 (RnD system, cat. no. MAB5869), HTRA2 (Cell Signaling Technology, cat. no. 2176S), β-actin (Sigma-Aldrich, cat no. A5441), phospho-p42/44MAPK Thr202/Tyr204 (Cell Signaling Technology, cat. no. 4376), p42/44MAPK (Cell Signaling Technology, cat. no. 9102), phospho-SMAD3 Ser-423/425 (Cell Signaling Technology, cat. no. 9520), SMAD3 (Cell Signaling Technology, cat. no. 9523). ALDH1A1 and β-actin were detected with mouse secondary antibodies conjugated to horseradish peroxidase (HRP). HTRA2, phospho-p42/44MAPK, p42/44MAPK, phospho-SMAD3 and SMAD3 were detected with anti-rabbit-hrp. Membranes were developed with West Pico ECL substrate. Chemi-luminescence signals were captured using UVP Imaging System.

### Transwell invasion assay

8 µm pore transwells sized to 24-well plates (Corning, cat. no. 3464), were coated with Growth Factor Reduced Matrigel (BD Biosciences, cat. no. 356230) diluted 1/10 in PBS for 30 min at 37°C. 75,000 cells were re-suspended in 200 µl of serum-free medium and plated on top of each transwell. 400 µl of serum-free medium was pipetted at the bottom of each well, in the presence or absence of recombinant CCL2 (Peprotech, cat. no. 300-04). Cells were incubated at 37°C for 24 h, fixed in 10% neutral formalin buffer and stained with 0.1% Crystal Violet. Cells were removed from topside of the transwell by cotton swab. Four images per well were captured using an Motic AE31 inverted microscope at 10× magnification. Cell number was quantified by ImageJ.

### ELISA

40,000 cells were seeded per well in 24-well plates for 24 h. Cells were incubated with 500 µl of complete media for 24 h. Conditioned medium was assayed for human CCL2 (Peprotech, cat. no. 900-K31) according to commercial protocols. Reactions were stopped using 2 M HCl. Absorbance was read at OD450 nm using a Biotek plate reader.

### Statistical analysis

Samples were plated in triplicate per group. All experiments were performed a minimum of three times. Data are expressed as mean±s.e.m. Statistical analysis was performed on Graphpad Software, using two-tailed *t*-test for two groups or one-way ANOVA with Bonferroni post-hoc analysis with more than two groups. Significance was determined by *P<0.05, **P<0.01, ***P<0.0001; n.s, not significant or *P*>0.05.

## Supplementary Material

Supplementary information
