# Supplementary material for: Role of ALDH1A1 and HTRA2 expression in CCL2/CCR2-mediated breast cancer cell growth and invasion
Source: Biol Open. 2019 Jun 17;8(7):bio040873. doi: 10.1242/bio.040873 (PMC6679398; doi:10.1242/bio.040873)
Supplement: Supplementary information [file biolopen-8-040873-s1.pdf]

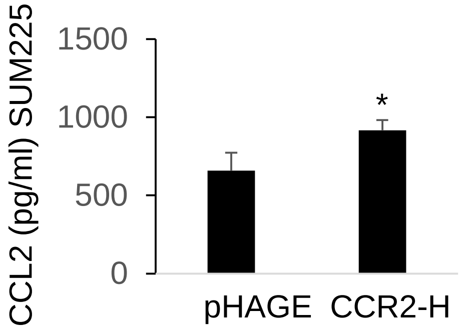

Figure S1. Effect of CCR2 overexpression on CCL2 expression. Conditioned medium from pHAGE or CCR2-H SUM225 cells were analyzed for CCL2 expression by ELISA. Statistical analysis was performed by Two Tailed T-test. Statistical significance was determined by *$p<0.05$. Mean±SEM are shown.

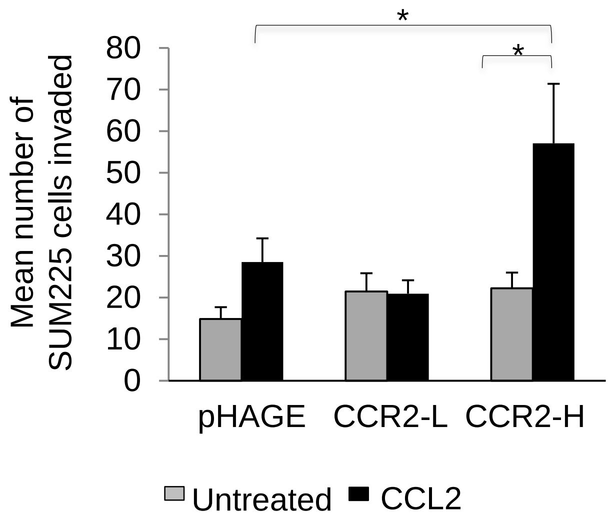

Figure S2. CCL2 enhances transwell invasion of CCR2 overexpressing SUM225 cells. phAGE control or CCR2 overexpressing SUM225 breast cancer cells were treated with 100 ng/ml CCL2 and analyzed for invasion through Matrigel coated transwells. Statistical analysis was performed using One Way ANOVA with Bonferroni post-hoc comparison. Statistical significance was determined by $p<0.05$. *$p<0.05$. Mean $\pm$SEM are shown.

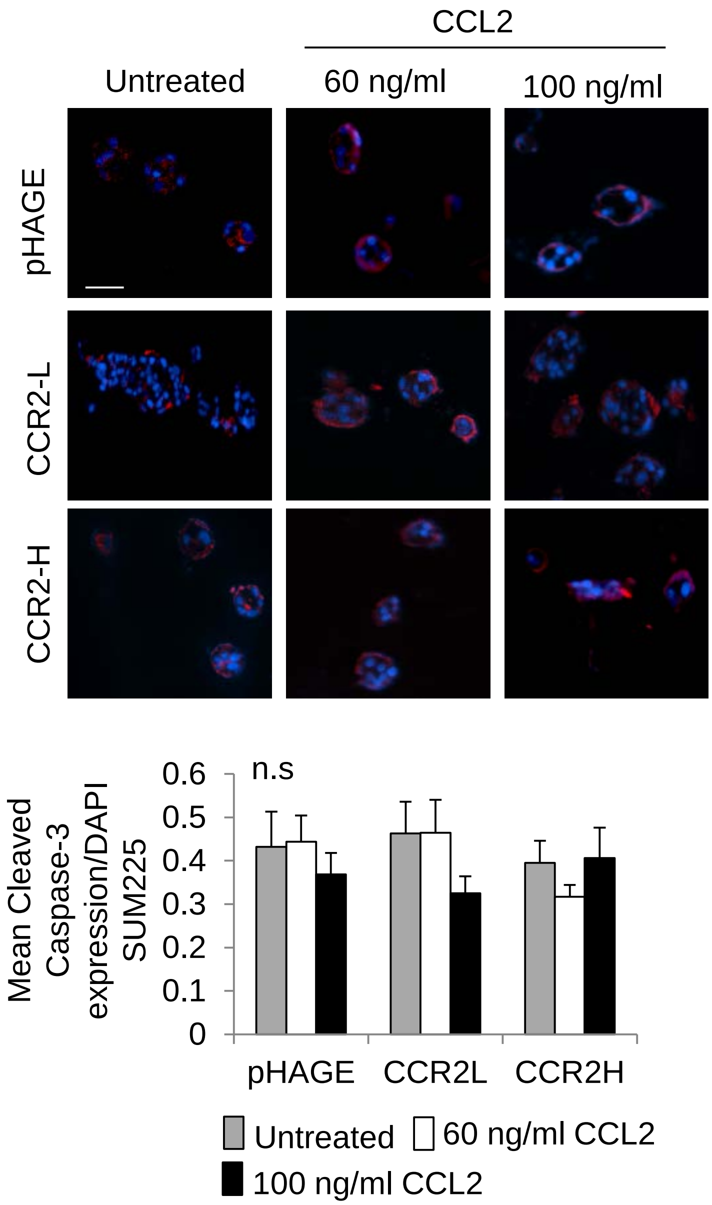

Figure S3. CCR2 overexpression in SUM225 cells does not affect apoptosis. SUM225 spheroids were immunofluorescence stained for Cleaved caspase-3 expression. Expression was quantified by Image J, and normalized to DAPI. Statistical analysis was performed using One Way ANOVA with Bonferroni post-hoc comparison. Statistical significance was determined by $p<0.05$. ns.=not significant. Scale bar=100 microns. Mean+SEM are shown.

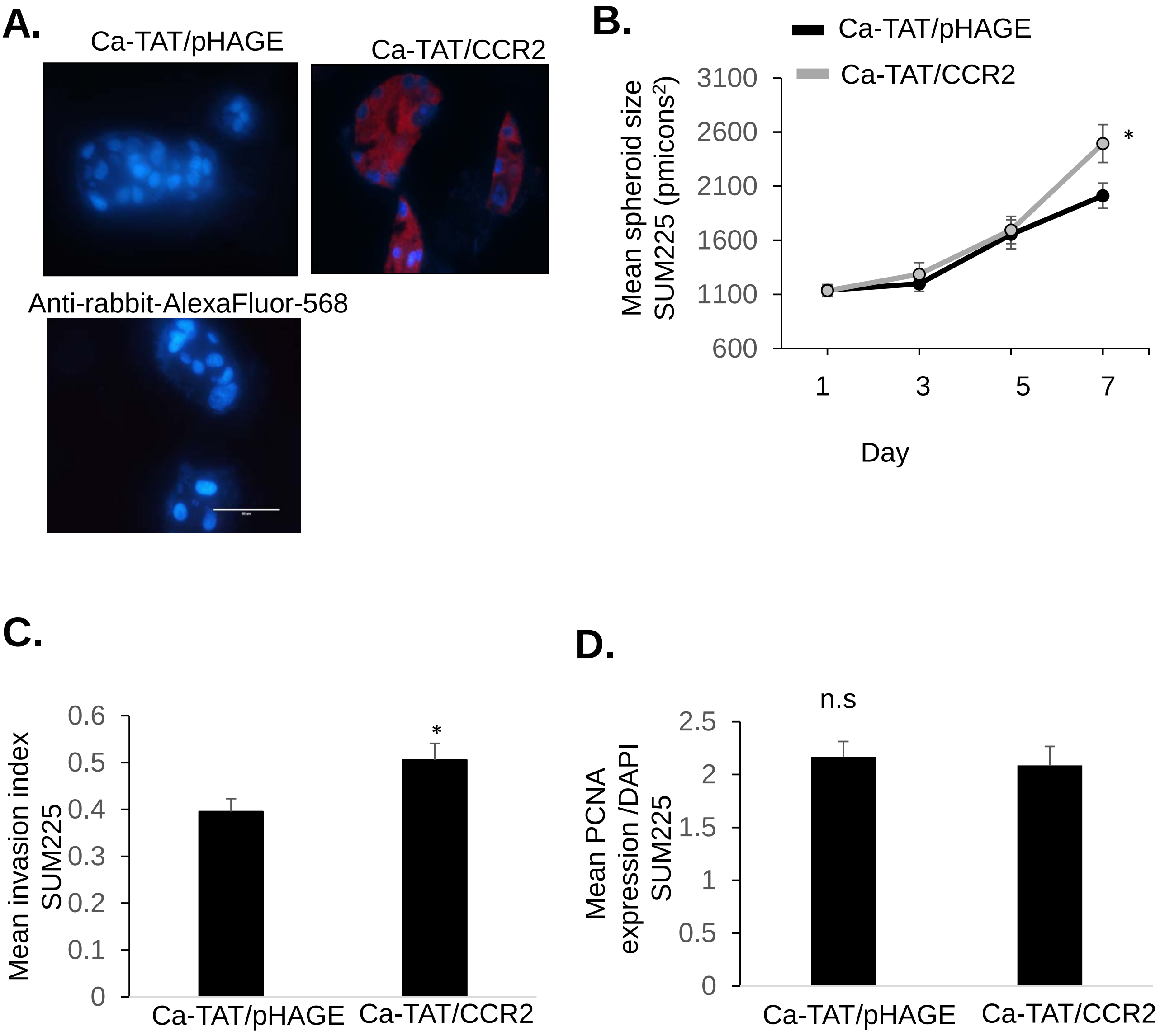

Figure S4. Ca-TAT mediated induction of CCR2 expression enhances growth ofSUM225 spheroids. SUM225 breast cancer cells spheroids were seed in Matrigel:Collagen. After 2 days, cells were transfected with Ca-TAT peptides complexed to pHAGE vehicle control or CCR2 overexpression plasmid, and cultured for up to an additional 5 days. Cultures were analyzed by **A.** immunofluorescence staining for CCR2 expression, **B.** spheroid size over time, **C**. Invasion index and **D.** PCNA expression by IF staining. Spheroid size and invasion index are normalized to sphere number. Expression was quantified by Image J, and normalized to DAPI/sphere. Mean number ± SEM spheroids analyzed: Ca-TAT/pHAGE= 78±25, Ca-TAT/CCR2=79±18. Statistical analysis was performed using Two Tailed T-test. Statistical significance was determined by $p<0.05$. *$p<0.05$, n.s= not significant. Scale bar=50 microns. Mean ±SEM are shown.

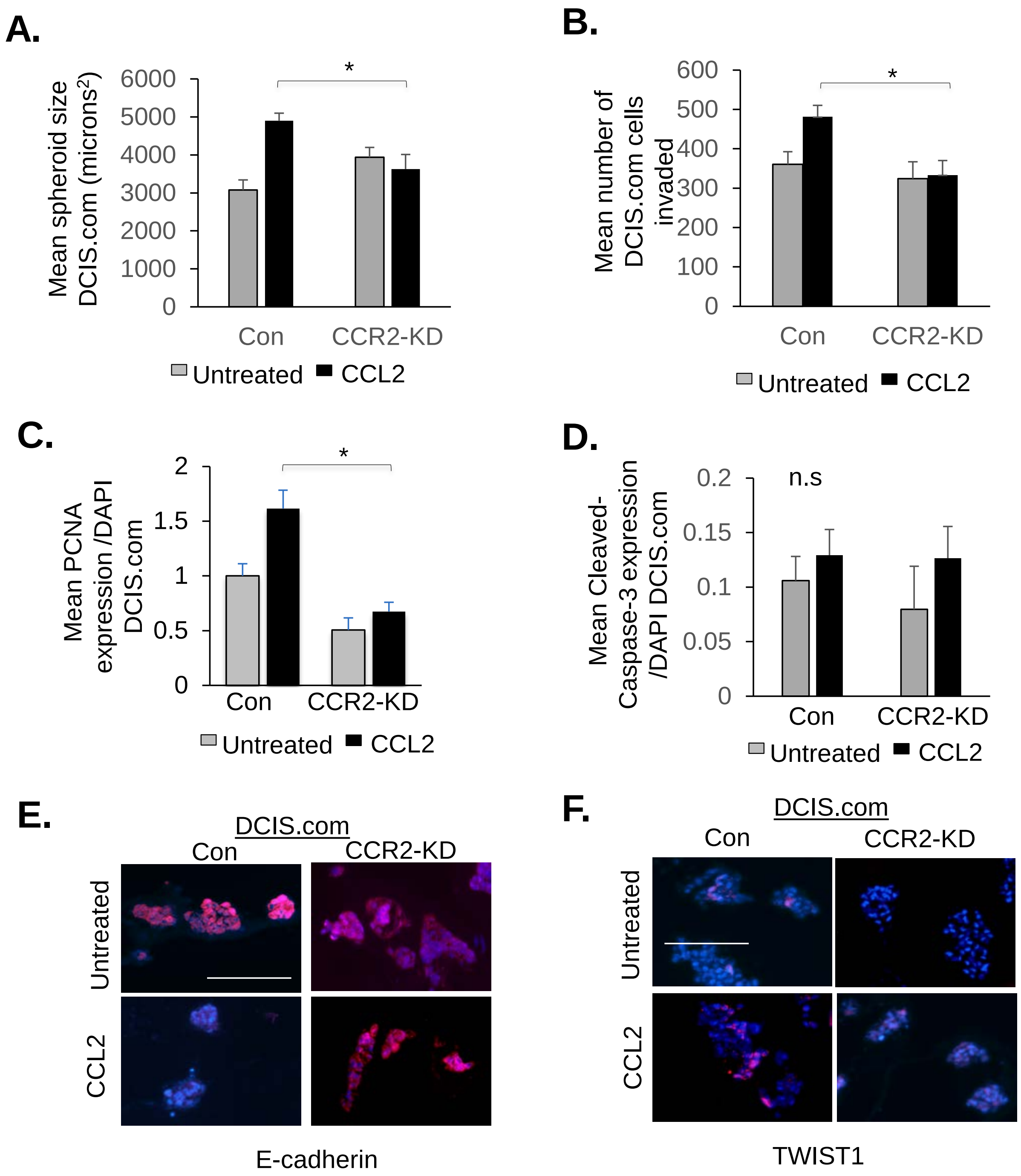

Figure S5. Stable CCR2 shRNA expression in DCIS.com cells inhibits growth and invasion. DCIS.com cells expressing Control (Con) CCR2 shRNAs (CCR2-KD) were treated with or without 60 ng/ml CCL2 and analyzed for **A.** growth in Matrigel: Collagen, **B.** transwell invasion, and expression of **C**. PCNA, **D**. Cleaved caspase-3, **E**. E-cadherin and **F.** TWIST1. Mean number of spheroids analyzed/group ±SEM: Con Untreated= 248±28, Con±CCL2=243±21, CCR2-KD Untreated=243±41, CCR2-KD±CCL2= 181+3. Expression was quantified by Image J, and normalized to DAPI. Statistical analysis was performed using One Way ANOVA with Bonferroni post-hoc comparison. Statistical significance was determined by $p<0.05$. Statistical significance was determined by $p<0.05$.
*$p<0.05$, n.s=not significant. Scale bar=100 microns. Mean±SEM are shown.

Biology Open • Supplementary information

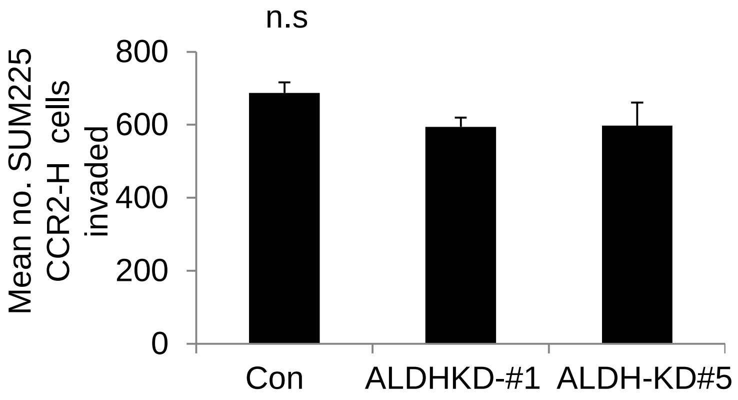

Figure S6. Effect of ALDH1 knockdown in SUM225 CCR2-H cells on transwell invasion. SUM225 CCR2-H cells stably expressing control shRNAs (Con) or ALDH1 shRNAs (ALDH1-KD#1, ALDH1-KD#5) were analyzed for transwell invasion. Statistical analysis was performed using One Way ANOVA with Bonferroni post-hoc comparison. Statistical significance was determined by $p<0.05$. n.s=not significant.

Biology Open • Supplementary information

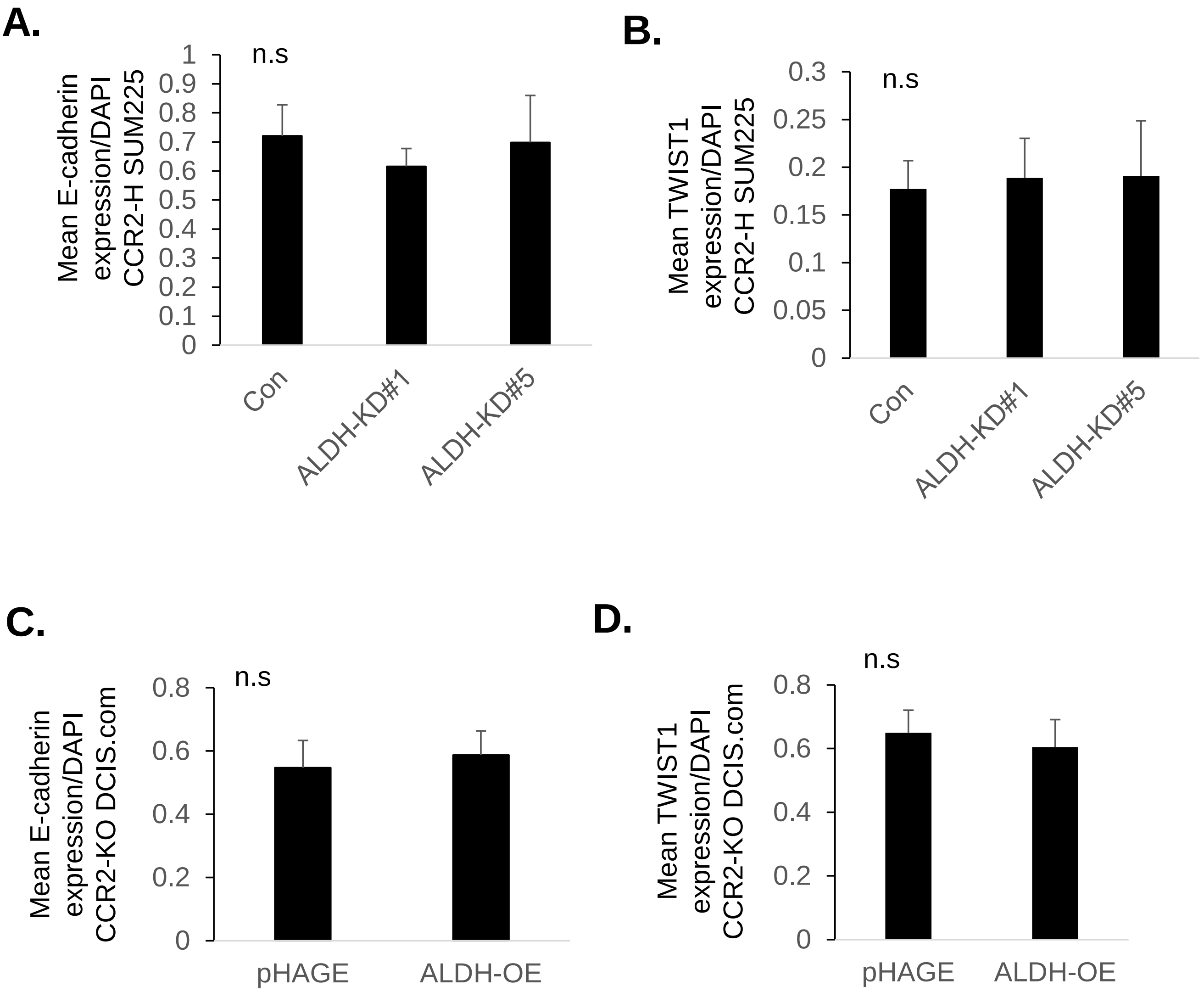

Figure S7. Effect of ALDH1A1 knockdown and overexpression on TWIST1 and E-cadherin expression. CCR2-H SUM225 cells expressing control shRNAs or shRNAs to ALDH1A1 wee cultured in 3D Matrigel: Collagen for 10 days, and were analyzed for expression of **A.** E-cadherin and **B.** TWIST1 by immunofluorescence staining. CCR2-KO DCIS.com cells expressing pHAGE control or ALDH1A1 were cultured in 3D Matrigel: Collagen for 10 days, and were analyzed for expression of **C.** E-cadherin and **D.** TWIST1 expression. Expression was quantified by Image J, and normalized to DAPI. Statistical analysis was performed using One Way ANOVA with Bonferroni post-hoc comparison (A,B) or Two Tailed T-test (C,D). Statistical significance was determined by $p<0.05$. Statistical significance was determined by $p<0.05$. n.s=not significant. Mean±SEM are shown.

Biology Open • Supplementary information

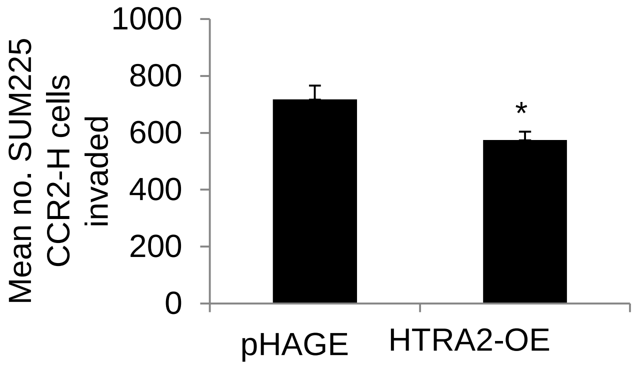

Figure S8. Effect of HTRA2 overexpression in SUM225 CCR2-H cells on transwell invasion. SUM225 CCR2-H cells stably expressing pHAGE vehicle control or overexpressing HTRA2 were analyzed for transwell invasion. Statistical analysis was performed using Two Tailed T-test. Statistical significance was determined by $p<0.05$. *$p<0.05$. Mean±SEM are shown.

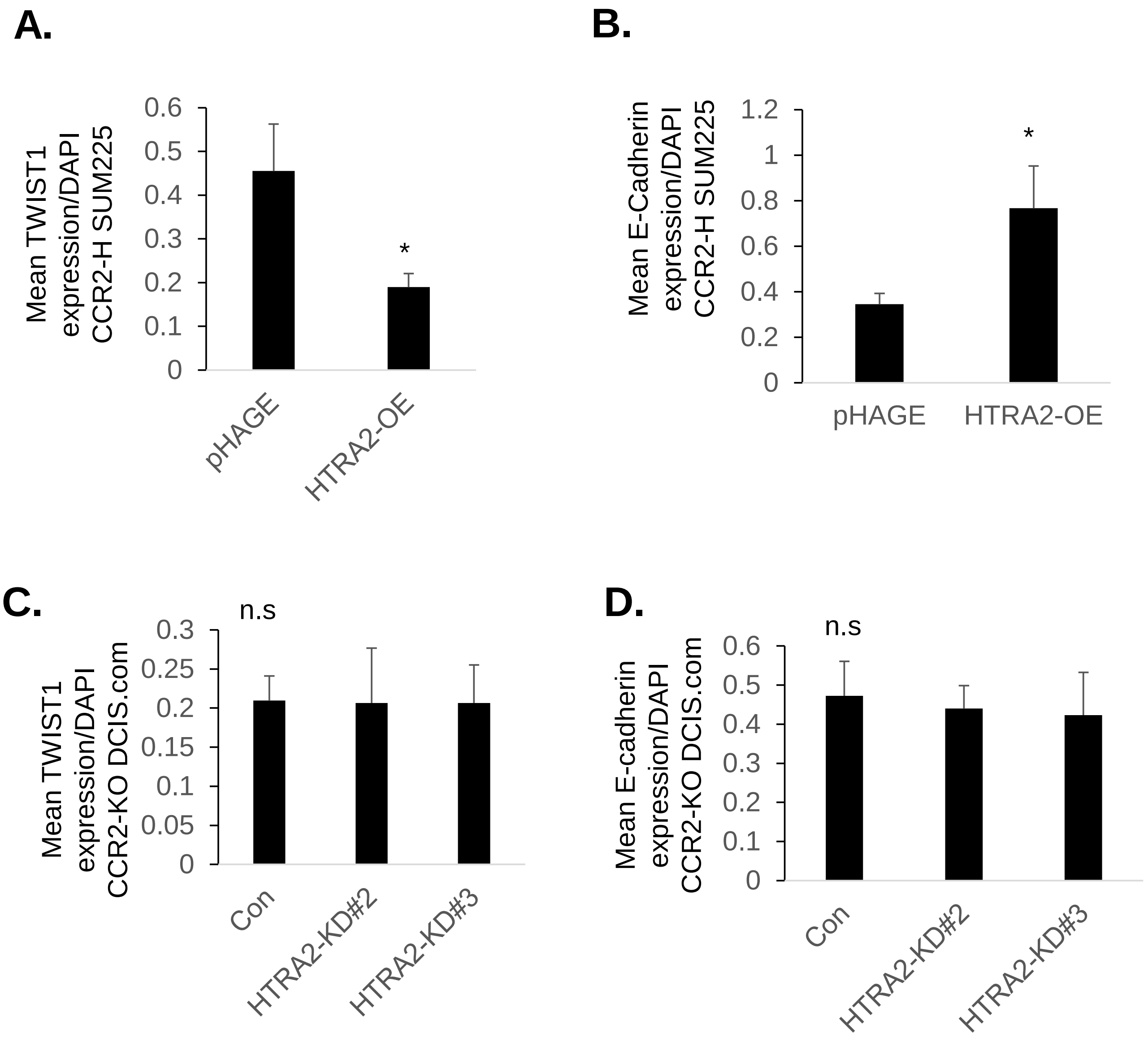

Figure S9. Effect of HTRA2 overexpression and knockdown on TWIST1 and E-cadherin expression. CCR2-H SUM225 cells expressing pHAGE control or HTRA2 were cultured in 3D Matrigel: Collagen for 10 days, and were analyzed for expression of **A.** TWIST1 and **B**. E-cadherin expression. CCR2-KO DCIS.com cells expressing control shRNAs or shRNAs to HTRA2 wee cultured in 3D Matrigel: Collagen for 10 days, and were analyzed for expression of **C.** TWIST1 and **D.** E-cadherin by immunofluorescence staining. Expression was quantified by Image J, and normalized to DAPI. Statistical analysis was performed using Two Tailed T-test (A,B) or One Way ANOVA with Bonferroni post-hoc comparison (C,D). Statistical significance was determined by $p<0.05$. *$p<0.05$, n.s=not significant. Mean±SEM are shown.

Biology Open • Supplementary information
